# The absence of the queuosine tRNA modification leads to pleiotropic phenotypes revealing perturbations of metal and oxidative stress homeostasis in *Escherichia coli* K12

**DOI:** 10.1093/mtomcs/mfac065

**Published:** 2022-09-09

**Authors:** Leticia Pollo-Oliveira, Nick K Davis, Intekhab Hossain, Peiying Ho, Yifeng Yuan, Pedro Salguero García, Cécile Pereira, Shane R Byrne, Jiapeng Leng, Melody Sze, Crysten E Blaby-Haas, Agnieszka Sekowska, Alvaro Montoya, Thomas Begley, Antoine Danchin, Daniel P Aalberts, Alexander Angerhofer, John Hunt, Ana Conesa, Peter C Dedon, Valérie de Crécy-Lagard

**Affiliations:** Department of Microbiology and Cell Science, University of Florida, Gainesville, FL 32611, USA; Department of Biological Engineering, Massachusetts Institute of Technology, Cambridge, MA 02139, USA; Department of Physics, Williams College, Williamstown, MA 01267, USA; Antimicrobial Resistance Interdisciplinary Research Group, Singapore-MIT Alliance for Research and Technology, Singapore 138602, Singapore; Department of Microbiology and Cell Science, University of Florida, Gainesville, FL 32611, USA; Department of Applied Statistics, Operations Research and Quality, Universitat Politècnica de València, Valencia 46022, Spain; Department of Microbiology and Cell Science, University of Florida, Gainesville, FL 32611, USA; Department of Biological Engineering, Massachusetts Institute of Technology, Cambridge, MA 02139, USA; Department of Biological Engineering, Massachusetts Institute of Technology, Cambridge, MA 02139, USA; Department of Microbiology and Cell Science, University of Florida, Gainesville, FL 32611, USA; Department of Microbiology and Cell Science, University of Florida, Gainesville, FL 32611, USA; Kodikos Labs, 23 rue Baldassini, Lyon 69007, France; Department of Chemistry, University of Florida, Gainesville, FL 32611, USA; The RNA Institute and Department of Biology, University at Albany, Albany, NY 12222, USA; Kodikos Labs, 23 rue Baldassini, Lyon 69007, France; School of Biomedical Sciences, Li Kashing Faculty of Medicine, University of Hong Kong, Pokfulam, SAR Hong Kong; Department of Physics, Williams College, Williamstown, MA 01267, USA; Department of Chemistry, University of Florida, Gainesville, FL 32611, USA; Department of Biological Sciences, Columbia University, New York, NY 10024, USA; Department of Microbiology and Cell Science, University of Florida, Gainesville, FL 32611, USA; Institute for Integrative Systems Biology, Spanish National Research Council, Paterna 46980, Spain; Department of Biological Engineering, Massachusetts Institute of Technology, Cambridge, MA 02139, USA; Antimicrobial Resistance Interdisciplinary Research Group, Singapore-MIT Alliance for Research and Technology, Singapore 138602, Singapore; Department of Microbiology and Cell Science, University of Florida, Gainesville, FL 32611, USA; Genetic Institute, University of Florida, Gainesville, FL 32611, USA

**Keywords:** iron-sulfur cluster, metal, nickel, oxidative stress, queuosine, tRNA modification

## Abstract

Queuosine (Q) is a conserved hypermodification of the wobble base of tRNA containing GUN anticodons but the physiological consequences of Q deficiency are poorly understood in bacteria. This work combines transcriptomic, proteomic and physiological studies to characterize a Q-deficient *Escherichia coli* K12 MG1655 mutant. The absence of Q led to an increased resistance to nickel and cobalt, and to an increased sensitivity to cadmium, compared to the wild-type (WT) strain. Transcriptomic analysis of the WT and Q-deficient strains, grown in the presence and absence of nickel, revealed that the nickel transporter genes (*nikABCDE*) are downregulated in the Q^–^ mutant, even when nickel is not added. This mutant is therefore primed to resist to high nickel levels. Downstream analysis of the transcriptomic data suggested that the absence of Q triggers an atypical oxidative stress response, confirmed by the detection of slightly elevated reactive oxygen species (ROS) levels in the mutant, increased sensitivity to hydrogen peroxide and paraquat, and a subtle growth phenotype in a strain prone to accumulation of ROS.

## Graphical Abstract

**Graphical Abstract:**
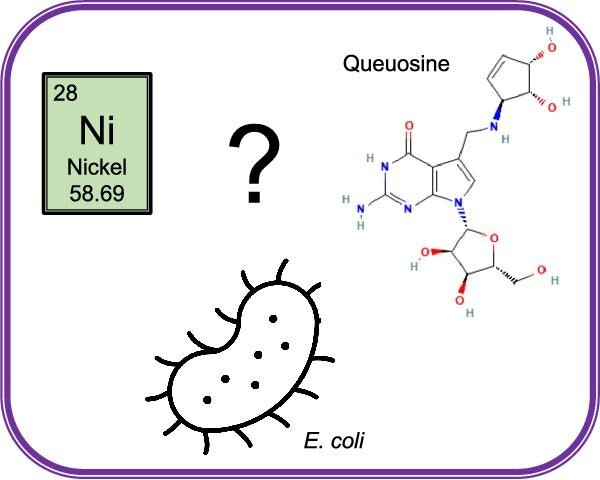
How does queuosine affect metal homeostasis in *E. coli*?

## Introduction

**Fig. 1:**
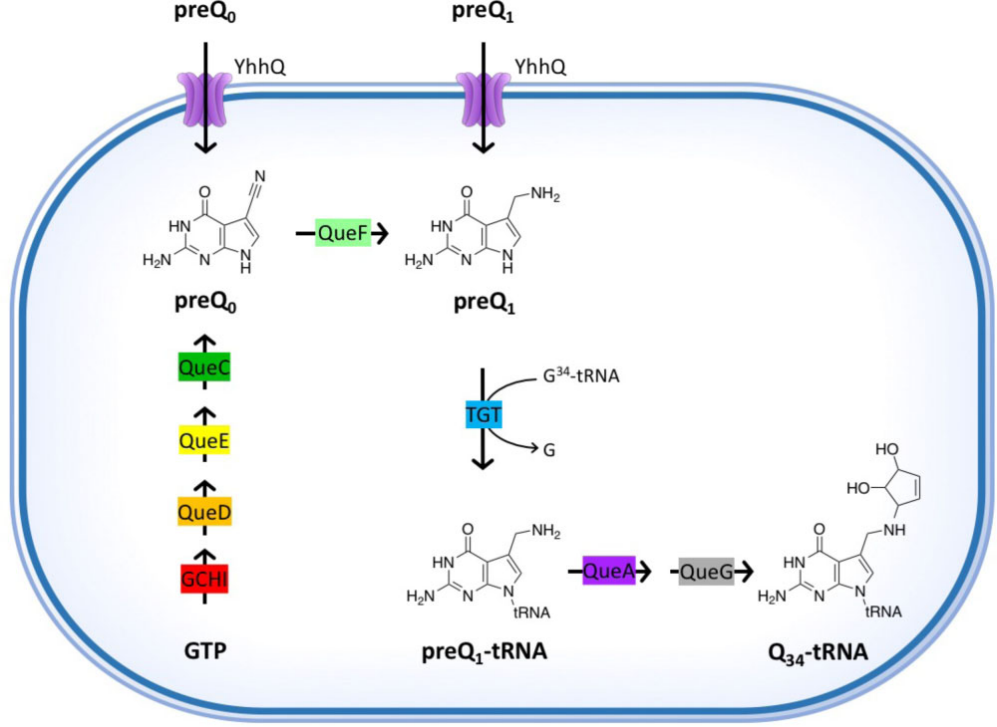
Diagram of the Q synthesis and salvage pathways. Biosynthesis of the Q modification at position 34 (Q_34_-tRNA) and preQ_0_/preQ_1_ salvage pathway in *E. coli*. Molecule abbreviations and protein names are described in the main text.

Queuosine (Q) is a structurally complex modification of transfer RNAs (tRNAs) found at position 34, the wobble position, of the tRNA anticodon-stem-loop (ASL). Q modifies the guanosine at G_34_U_35_N_36_ anticodons of tRNAs that decode the dual synonymous codons NAU and NAC (U- and C-ending codons), corresponding to the amino acids Asn, Asp, His, and Tyr.^1^ Found in Bacteria and Eukaryotes, the Q precursor is only synthesized by the former, while mammals need to salvage the Q-base queuine (q) from the diet and gut microbiota, giving q the status of a micronutrient.^2–5^ In bacteria, the biosynthesis of the Q precursor 7-aminomethyl-7-deazaguanine (preQ_1_) from guanosine triphosphate (GTP) consists of five enzymatic steps (Fig. 1), with the participation of FolE (GCHI), QueD, QueE, QueC, and QueF enzymes, respectively.^6^ The tRNA guanine transglycosylase (TGT) enzyme then inserts preQ_1_ into the wobble position of the targeted tRNA, with a concomitant displacement of the guanine base.^7^ TGT is therefore considered the signature enzyme for Q formation. Two additional enzymatic steps, performed by QueA and QueG (or QueH), mature the inserted preQ_1_ to the final Q-modified tRNA^8–10^ (Fig. 1). Some bacteria are also capable of salvaging Q precursors. In *Escherichia coli*, the YhhQ transporter imports both preQ_0_ and preQ_1_ precursors from the environment^11^ (Fig. 1), and other bacteria, particularly mammalian pathogens, can also salvage q.^12^

The recognition between the mRNA codon and the tRNA anticodon follows the canonical Watson-Crick base pairing (A: U, U: A, G: C, C: G) for the first two positions of the codon triplet (with the 3^rd^ and 2^nd^ bases of the anticodon), while the pairing of the 3^rd^ base of the codon with the 1^st^ base of the anticodon (tRNA wobble position 34) follows non-canonical rules for base pairing. Such a wobble explains the degeneracy of the genetic code, since a particular tRNA can decode more than one synonymous codon.^13^ The most chemically complex modified nucleosides are found in the ASL, at positions 34 and 37, influencing translational efficiency and fidelity.^14,15^ As a modification of position 34, the effects of Q on protein translation have been extensively studied *in silico, in vitro*, and *in vivo* in different organisms, with conflicting and complex findings. The presence of Q generally reduces biases between C- or U- ending codons but in different directions and at different codons.^16–22^ Q modification also improves translational fidelity. The suppression of errors occurs in a complex and context-dependent way, varying with the organism and tRNA species.^20,23^

If progress has been made on how Q affects translation, its physiological importance has remained elusive particularly as it was repeatedly lost during evolution.^24^ In *E. coli*, the absence of Q is not critical in exponential growth but reduces cell viability in stationary phase.^25^ In a handful of other bacteria, a variety of phenotypes for Q deficiency have been reported such as sensitivity to oxidative stress,^26^ defects in virulence,^27,28^ and symbiosis,^29^ but the molecular basis for these phenotypes remains unknown.

One theme that has been recurrent throughout the years is the link between Q and Q biosynthetic genes to metal metabolism. In *Drosophila melanogaster*, Q increases resistance to cadmium,^30^ which is in accordance with the induction of the queuine tRNA-ribosyltransferase under acute cadmium stress in the scallop *Patinopecten yessoensis*.^31^ The overexpression of QueC (ALU1-P) from *Arthrobacter viscosus* confers aluminum resistance to *E. coli*.^32^ The *queC* gene was also upregulated during a nickel challenge in Archaea.^33^ In the presence of copper, *queE* and *yhhQ* expression was induced in the bacterium *Erwinia amylovora*,^34^ while in *Acinetobacter baumannii*, QueD and Tgt become more cell abundant during metal starvation induced by calciprotein, an extracellular metal-sequestering protein.^35^ Further evidence of this link with metal homeostasis comes from the Q biosynthetic pathway, with most of the enzymes being metal dependent: FolE1, QueD, QueC, and TGT require zinc for activity,^36–39^ while QueE, QueG, and QueH require iron.^10,40^,^41^ It is noteworthy that a comparative analysis of the Zur regulon revealed that certain bacteria contained two copies of the *queD* gene (*queD* and *queD2*), with *queD2* predicted to be under control of the negative regulator Zur, whose regulon is activated under low zinc.^42,43^

Based on the evidence for an involvement of Q in metal homeostasis, we tested the sensitivity of the *tgt* mutant (Q^–^ strain in *E. coli* MG1655 background) to different metals and found varied pleiotropic phenotypes that were further explored by a combination of transcriptomics, proteomics, and physiological studies suggesting that the absence of Q leads to an atypical oxidative stress response.

## Methods

### Media and plasmids

Luria-Bertani (LB) broth and agar (tryptone 10 g/l, yeast extract 5 g/l, sodium chloride 10 g/l, Fisher Scientific BP1426-2 and BP1425-2) were routinely used for growth of *E. coli* cells at 37°C from frozen stocks, and for some of the phenotypic assays. When required, 50 μg/ml of kanamycin (Kan) was added to the medium. M9 minimal medium (12.8 g/l Na_2_HPO_4_·7H_2_O, 3.0 g/l KH_2_PO_4_, 0.5 g/l NaCl, 1.0 g/l NH_4_Cl, 2.0 mM MgSO_4_, 0.1 mM CaCl_2_) was used in motility assays, with the appropriate amount of agar. Low-phosphate (LP) medium was adapted from^44^ and used in cadmium sensitivity tests. LP composition in this study was 6.06 g/l Tris (Amresco), 0.3% casamino acids, 4.68 g/l NaCl, 1.49 g/l KCl, 1.07 g/l NH_4_Cl, 0.2 g/l MgCl_2_, 0.03 g/l CaCl_2_·6H_2_O, 0.172 g/l Na_2_HPO_4_·7H_2_O (all Fisher Scientific), 0.43 g/l Na_2_SO_4_, 5 mg/l ammonium iron (III) citrate (CAF) (Sigma Aldrich), and 1 ml/l of SL7 trace element solution, which contains in 1 l: 1 ml of 25% HCl, 60 mg H_3_BO_3_ (Fisher Scientific), 70 mg ZnCl_2_, 100 mg MnCl_2_·4H_2_O, 20 mg CuCl_2_·2H_2_O, 20 mg NiCl_2_·6H_2_O, 40 mg NaMoO_4_·2H_2_O (all Sigma Aldrich) and 200 mg CoCl_2_·6H_2_O (Alfa Aesar). Glycerol at 0.2% was used as carbon source. MA medium, a pH 7.0 MOPS-based medium used for iron-related sensitivity tests, was composed by 100 mM MOPS, 10 mM NaH_2_PO_4_, 11 mM K_2_HPO_4_, 27 mM (NH_4_)SO_4_, 1.825 mM MgSO_4_·7H_2_O, 34 mM CaCl_2_·2H_2_O, and micronutrients (NH_4_)_6_(Mo_7_O_24_)·4H_2_O, 3 × 10^–6^ mM; H_3_BO_3_, 4 × 10^–4^ mM; CoCl_2_, 3 × 10^–5^ mM; CuSO_4_, 10^–5^ mM; MnCl_2_, 8 × 10^–5^ mM; ZnSO_4_, 10^–5^ mM. The medium was freshly supplemented with 0.001 g/l ammonium iron (III) citrate (CAF) as iron source and with 0.2% glycerol as carbon source.

### Strains and plasmids

All strains used in this study are listed in Table S1 (Supplemental file 1), and all oligonucleotide primers are listed in Table S2 (Supplemental file 1).

*Escherichia coli* K-12 MG1655 was used as the WT strain. *Escherichia coli* Δ*tgt* construction (VDC4561) was obtained by classical P1 transduction^45^ of the *tgt::kan* allele from strain VCD4548, whose gene disruption was created by recombination^46^ using the knockout primers CH316 and CH317. New *tgt* mutant strains were created by P1 transduction of the *tgt::kan* allele from strain VCD4561 into MG1655, generating strains LPO0126, LPO0127, and LPO0128, which were used for the FNR and Fur Western blots, for the EPR experiments, and also to confirm the metal and other phenotypes. The strain VDC4560 was generated exactly like VDC4561 but accidently lost the *lacZ* gene during this process and was used as a negative control for the beta-galactosidase assays. The Δ*queD* Δ*tgt* strains (LPO0120-LPO1025) were obtained by P1 transduction of the *tgt::kan* allele from strain VCD4561 into a Δ*queD* strain with no antibiotic resistance cassette. For the construction of Δ*yhhQ* Δ*queD* Δ*tgt* (VDC4584), the strain Δ*yhhQ* Δ*queD* (VDC4578)^11^ had the kanamycin resistance cassette removed by Flp-catalyzed excision^47^ and the *tgt::kan* allele inserted by P1 transduction from VDC4561. The strains used to measure the activity of promoters regulated by Fe-S clusters P*iscR::lacZ* (DV901), P*hmpA::lacZ* (DV1301), and P*ydfZ::lacZ* (BR616) were kindly provided by Frédéric Barras. The strain used to measure the activity of the *ryhB* promoter P*ryhB::lacZ* (PM2230) was a gift from Pierre Mandin, and was constructed by amplifying the P*ryhB* promoter with oligonucleotides PryhB-300-F and PryhB-lacZ-R using *E. coli* strain MG1655 as a template, and the PCR product was then recombined in strain PM1205, as described in.^48^ The *tgt* gene was deleted from these strains by P1 transduction of the *tgt::kan* allele from strain VCD4561.

The Hpx^–^ strain was a kind gift from James Imlay^49^ and the *tgt::cat* allele was P1 transducted into the Hpx^–^ and MG1655 strains. The deletion construct was made using the linear recombination method described by Datsenko and Wanner.^46^ Deletion primers (YY190 and YY191) were designed with 50 bp of homology to the gene to be deleted. The P1 or P2 sites were added to the 3’ end of each primer to amplify the chloramphenicol resistance cassettes from pKD3. PCR products were cleaned using the Zymo Clean and Concentrator-5 kit (Zymo Research), and 200 ng of PCR product was transformed via electroporation into freshly prepared electrocompetent MG1655 carrying the temperature-sensitive pKD46. Chloramphenicol resistant isolates were selected on LB-agar plates with 30 μg/ml of chloramphenicol. All mutations were verified by PCR for loss of the target gene and presence of the antibiotic resistance marker in the correct locations.

For complementation assays using the overexpression of the *tgt* gene in trans, the *tgt* gene was amplified from WT *E. coli* MG1655 strain and cloned into pBAD24 plasmid, resulting in the pTGT plasmid.^50^ GC10 Chemically Competent cells (Gene Choice, Genesee Scientific) were used for routine cloning. *Escherichia coli* WT and Δ*tgt* chemically competent cells were freshly transformed for each assay and selected with 100 μg/ml ampicillin.

## Phenotypic analysis

### Growth curves in Bioscreen C Analyzer

*Escherichia coli* WT and *tgt* mutant strains, in three to six biological replicates, were diluted from overnight saturated cultures to optical density (OD_600_) of 0.005, in a final volume of 200 μl of appropriate medium. Growth at 37°C with continuous shaking was monitored by measuring OD_600_ every 30 min for 24 or 48 h in a Bioscreen-C Automated Growth Curve Analysis System (Growth Curves USA, MA, USA). Chemical sensitivities were tested in appropriate medium using 0.5–3.0 mM NiCl_2_·6H_2_O (Sigma Aldrich), 0.5–1.25 mM CoCl_2_·6H_2_O (Alfa Aesar, Puratronic), 4–10 μg/ml streptomycin sulfate (Strep, Sigma Aldrich), 10–15 μg/ml spectinomycin dihydrochloride pentahydrate (Spec, Fluka, BioChemiKa), 5–20 μg/ml ampicillin sodium (Amp, Acros), 1–5 μg/ml gentamycin sulfate (Gm, Sigma Aldrich), 0.9–1.5 mM hydrogen peroxide (Fisher Scientific), and 650–900 μM methyl viologen (Sigma Aldrich).

For complementation of the Δ*tgt* phenotypes, the overexpression of the *tgt* gene from pTGT was induced with 0.02% of arabinose. The empty plasmid pBAD24 was used as a negative control, and for plasmid maintenance the medium was supplemented with 100 μg/ml ampicillin.

### Plate assays

Nickel sensitivity assay was performed in LB medium supplemented with 2 mM NiCl_2_·6H_2_O (Sigma Aldrich) and cadmium sensitivity assay in LP medium supplemented with 25 μM CdSO_4_·xH_2_O (Sigma Aldrich). Streptonigrin (SNG) sensitivity was tested in MA medium added by 0.75 μg/ml SNG (Sigma Aldrich). Attenuation of the streptomycin (Strep) phenotype upon addition of an iron chelator was assayed in MA medium supplemented with 2 μg/ml streptomycin in the presence of an iron source (0.001 g/l CAF) or in the absence of an iron source by addition of 100 μM 2,2′-Bipyridyl (Sigma Aldrich). Sensitivity to methyl methane sulfonate (MMS) was performed in LB medium supplemented with 0.045%–0.065% MMS. For all plate assays, overnight saturated cultures were diluted to OD_600_ 0.2 in the assay medium and grown for about 5 h, when cell cultures reached optical density around 1.0, cultures were normalized to the same OD and 10-fold serially diluted. Seven microliters of each dilution was plated onto appropriate solid media. For complementation of the Δ*tgt* phenotypes, the overexpression of the *tgt* gene from pTGT was induced with 0.02% of arabinose. For plasmid maintenance, the medium was supplemented with 100 μg/ml ampicillin.

## Transcriptomics

### Sample preparation and sequencing

Three biological replicates of *E. coli* MG1655 WT and Δ*tgt* strains were grown overnight in LB medium, and saturated cultures were diluted to OD_600_ 0.05 in 20 ml of LB only and in 20 ml of LB medium added by 2 mM NiCl_2_·6H_2_O (Sigma Aldrich). Cells were incubated at 37°C with continuous shaking at 200 rpm and harvested during exponential phase (OD_600_ 0.6–0.8) for RNA isolation. RNA was stabilized with RNAprotect Bacteria Reagent (Qiagen #76506) and isolated using the RNeasy Mini Kit (Qiagen # 74104). Remaining cell pellets were stored at –80°C for further proteomics analysis. RNAseq sample preparation and sequencing was performed by Girihlet Non-Canonical Genomics (Oakland, CA—https://www.girihlet.com/). Briefly, the quality and quantity of total RNA was evaluated using the Agilent RNA 6000 Nano Kit on an Agilent Bioanalyzer. rRNA was removed using Ribo-Zero Magnetic Kit* (Gram-Negative Bacteria) Epicentre. The RNA sequencing library was prepared with TruSeq RNA Library Prep Kit v2 (Illumina). Libraries were analyzed on a Bioanalzyer for average base pair distribution and quantified using Qubit Fluorometric Quantitation (Thermo Fisher Scientific). Sequencing was performed in the HiSeq 2500 System (Illumina) with 75 bp Paired End sequencing and 40 million reads per sample.

### RNAseq data analysis

The quality of the raw RNA-seq data was evaluated with the FastQC tool,^51^ and Trimmomatic^52^ was applied to remove the adaptors sequences at the beginning of the reads. The *E. coli* strain K-12 MG1655 genome fasta and gff files (GCF_000005845.2_ASM584v2_genomic) were downloaded from the National Center for Biotechnology Information website (https://www.ncbi.nlm.nih.gov/). The genome was indexed using Bowtie2,^53^ and the mapping was performed using TopHat.^54^ Read counts were obtained using HTSeq count script^55^ and normalized considering transcript length and sequencing depth to obtain RPKM.^56^ The ARSyN method^57^ was applied to remove batch effects, and effective batch effect removal was checked by principal component analysis. Differential expression was assessed using the Limma package, applying the Voom transformation.^58^ Significant differentially expressed genes were called at an false discovery rate (FDR) < 0.05. All scripts are available at https://github.com/vdclab/RNAseq-analysis. Gene IDs were obtained from EcoGene 3.0 (ecogene.org, accessed on December 2017)^59^ and Gene Ontology (GO) term annotations (Gene Ontology Consortium, 2021) were obtained from the EcoCyc database (ecocyc.org, accessed on December 2017).^60^ Raw data (fastq files) and processed data have been deposited in NCBI's Gene Expression Omnibus^61^ and are accessible through GEO Series accession number GSE181239.

## Proteomics analysis

### Protein isolation

tein extracts were prepared from the same batch cultures used for the transcriptomics analysis of the following samples: WT and *tgt* mutant strains grown in LB medium, in biological triplicates. Soluble protein was isolated from bacterial cells by harvesting at 200 × g for 10 min at 4°C and washing cell pellets with ice-cold phosphate-buffered saline (PBS) and frozen at –80°C. To prepare cell lysates, pellets were resuspended in 500 μl of lysis buffer (20 mM Na-phosphate, pH 6.8, 10 mM DTT, 1 mM EDTA, 0.1% v/v Tween, 1 mM PMSF, Roche protease inhibitor cocktail, 3 mg/ml lyticase, and 1.25 U/ml benzonase) and incubated at 30°C with mild shaking for 30 min. Glass beads were used to disrupt cells using a Precellys 24 disrupter; 2 cycles of 25 s at 6500 rpm; samples were kept on ice between each cycle. Cell lysates were then centrifuged for 20 min at 200 × g at 4°C and supernatant fractions were aspirated, analyzed by the bicinchoninic acid (BCA) method,^62^ and adjusted to equimolar protein concentrations [4.8 mg/ml for protein gels and 4 mg/ml for liquid chromatography–mass spectrometry (LC-MS)/MS analysis] across samples.

### Protein processing, labeling with isobaric tags, and peptide fractionation

Protein samples were aliquoted (100 μg per sample), dried by vacuum centrifugation, reconstituted in 100 mM TEAB and 10% acetonitrile (v/v) by bath sonication, and digested with trypsin in a 1:30 (w/w) ratio overnight at 37°C. Aliquots of resulting protein digests (from 100 μg of total protein) were then labeled with TMT 6-plex reagents according to the manufacturer's protocol. Labeled peptides (5-μl aliquots) from each biological replicate were combined to reconstitute a full 6-plex label set and subjected to preliminary qualitative analysis on a Thermo Scientific EASY-nLC 1200 interfaced to a Thermo Scientific Q Exactive Hybrid Quadrupole-Orbitrap mass spectrometer. Median total ion intensities for each label were calculated and used to normalize volumetric mixing of respective labels, so as to avoid signal suppression or bias from any one label. After combining labels into 6-plex sets, samples were desalted with C18 SpinTips (Protea), dried by vacuum centrifugation, and reconstituted in IPD buffer (Agilent) without glycerol. Isoelectric focusing was performed from pH 3 to 10 over 24 wells on an Agilent 3100 OFFGEL fractionator according to the manufacturer's protocol (OG24PE00). Each of the 24 fractions was collected, dried by vacuum centrifuge, resuspended in 0.1% formic acid in water, and analyzed by nano-LC-MS/MS.

### LC-MS analysis of the *E. coli* proteome

TMT proteomics experiments were performed on an Agilent 1200 nano-LC-Chip/MS interfaced to an Agilent 6550 iFunnel Q-TOF LC/MS. The HPLC-Chip configuration consisted of a 160-nL enrichment column and a 150 mm × 75 μm analytical column (G4340-62001 Zorbax 300SB-C18). The following mass-spectrometry grade mobile phases were used: 0.1% formic acid in water (solvent A), and 0.1% formic acid in acetonitrile (solvent B). A 130-min linear gradient was used for HPLC separation with 10 min for column washing and equilibration between runs. Samples (1–2 μl injections) were loaded onto the enrichment column at 3% (v/v) B at flow rates of 3 μl/min. The analytical gradient of solvent B was performed at a constant flow rate of 0.3 μl/min using the following solvent transitions on the nanoflow pump: 0–1 min, held at 1% (v/v); 1–10 min, 1–15%; 10–101 min, 15–35%; 101–121 min, 35–75%; 121–123 min, 75–98%; 123–126 min, held at 98%; 126–127 min, 98–1%; 127–130 min, held at 1%. The Q-TOF was operated at high sensitivity (4 GHz) in positive ion mode with the following source conditions: gas temperature 350 ºC, drying gas 13 l/min, fragmentor 360 V. Capillary voltage was manually adjusted between 1800 to 2150 V to maintain a steady nanospray plume. Data were acquired from 300 to 1700 *m/z* with an acquisition rate of 6 spectra/s in MS mode, and from 50 to 1700 *m/z* with an acquisition rate of 3 spectra/s in MS/MS mode. A peptide isotope model (charge state 2+) was used to detect a maximum 20 precursors per cycle at a minimum threshold of 25 000 counts/spectra at a narrow isolation window (∼1.3 *m/z).* Sloped collision energy (C.E.) was used to maximize collision induced dissociation of detected isobarically tagged peptides according to the following rules: charge state 2 + C.E. slope 4.2, offset 3.5; charge states ≥ 3 + C.E. slope 4.2, offset 4.

LC-MS data was extracted and evaluated for quality using the molecular feature extraction (MFE) algorithm in MassHunter Qualitative Analysis software (v B06.00). Test injections (3–4) from each fraction of the first technical replicate were used to optimize injection volumes for second and third biological replicates with the aim of maximizing the number of extracted molecules with peptide-like features. For each fraction, the MFE list of molecular ions was exported and used to exclude spectral acquisition of these ions in subsequent technical replicates. Each of the 24 fractions from biological triplicates were injected in technical duplicate—spectra generated from technical replicate #1 were acquired without use of an exclusion list, whereas spectra generated from technical replicates #2 were acquired with the exclusion list. Data from MassHunter Qualitative Analysis were exported to Mass Profiler Professional (v B03.00) for analysis of technical reproducibility. This process was repeated for all three biological replicates. Mass spectra were processed using Spectrum Mill (Agilent, v B06.00) and Scaffold Q+ (v Scaffold_4.8.8), and quantified protein associations were manually analyzed by binning and averaging peptide quantities across related protein groups in Excel.

### Gene-specific codon usage analysis

A codon counting algorithm^63^ was used to calculate the gene-specific codon usage frequencies for all genes in the *E. coli* genome using proteomics data. Over- or under-representation of a specific codon for a particular gene relative to the genome average was determined by calculating the average Z-score based on a hypergeometric distribution with a cut-off of *P* < 0.01. The upregulated and downregulated proteins in the *tgt* mutant relative to wild-type *E. coli* were selected based on the log_2_(*tgt*/WT) values, and codon usage analysis was performed for the filtered set of genes. Gene-specific codon usage data were visualized as a heat map using GraphPad Prism 9, as was multivariate statistical analysis. The principal components with the largest eigenvalues that together explain 75% of the total variance were selected for analysis.

### Western blot

For FNR and FUR protein detection by Western blot, WT and *tgt* mutant strains were grown in LB medium at 37°C, shaking at 200 rpm, and collected at OD_600_ of 0.6. Cells from 775 ml of each culture were collected by centrifugation at 3000 g, 30 min, 4°C, and pellets were stored at –80°C. Cell pellets were then resuspended in 10 ml of cold, fresh extraction buffer (50 mM Tris-Cl pH 7.4, 50 mM NaCl, 1 mM EDTA, 1 mM DTT, 1 mini tablet of Pierce Protease Inhibitor EDTA-free cat #88666) and disrupted by passage for three cycles through a French Press at 2000 psi. After centrifugation (3000 g, 20 min, 4°C) to remove cell debris, the soluble and insoluble fractions were obtained by ultracentrifugation at 208 000 g for 1 h 45 min at 4°C (Beckman Type 70 Ti rotor). The remaining insoluble material (pellet) was resuspended in 1 ml of 8 M urea, sonicated for three cycles of 10 s at intensity 7, with 20 s interval, and centrifuged at 25 000 g, 4°C, 10 min. Protein concentration in the soluble fraction was determined with the Pierce BCA Protein Assay Kit (Thermo Scientific) using bovine serum albumin as standard. Diluted in SDS loading buffer (10% SDS, 250 mM Tris-Cl pH 6.8, 500 mM DTT, 25% glycerol, bromophenol blue), 20 μg of the soluble fraction from each strain, and proportionally the same amount of insoluble fraction, were resolved in a 18% acrylamide gel. For Western blot, proteins were transferred to a PVDF membrane, probed with the primary antibody (1:10 000 rabbit anti-FNR or 1:5000 rabbit anti-FUR), detected with 1:5000 goat anti-rabbit IgG (H + L) Poly-HRP Secondary Antibody, HRP (Thermo Fisher Scientific, # 32260) and revealed using Clarity Western ECL Substrate (Bio-Rad, #1705061). The anti-FNR antibody and the anti-FUR antibody were kindly provided by Erin Mettert^64^ and Michael Vasil,^65^ respectively.

### Detection of ROS levels

Levels of reactive oxygen species (ROS) were measured in the *E. coli* WT and *tgt* mutant strains using flow cytometry coupled with the probe CellROX Green according to.^66^ Briefly, four to six biological replicates of each *E. coli* strain were grown to mid-log phase (OD_600_ 0.4–0.8) in LB, centrifuged at 3500 × g for 7 min and resuspended in 10% LB (diluted with PBS) to an OD_600_ of 0.1 before treatment was performed in 96-well plates. Each well, with a total volume of 200 μl, contained bacteria (final OD_600_ of 0.01) in 10% LB, 0.5 μM CellROX Green, with or without treatment of 200 μM menadione. The plate was incubated for 1 h at 37 °C before staining for nucleic acids with DAPI (5 μg/ml) and fixation in 4% paraformaldehyde at 25 °C followed by analysis with flow cytometry.

For flow cytometric analysis, cells were run on a LSRII flow cytometer (BD Biosciences) within 2 h using a HTS fluidics system. CellROX Green was excited with an argon laser and detected by a 530/30 nm band-pass emission filter, while DAPI was excited with a UV laser and detected by a 450/50 nm band-pass emission filter. A total of 50 000–100 000 events were collected for each sample via Diva (BD Biosciences) and analyzed on FlowJo v10.0.6 (Tree Star, Inc.). Forward-scatter (FSC; correlates with cell size) and side-scatter (SSC; correlates with cellular granularity) of light was used to gate bacterial samples using unstained cells. DNA-positive cells were then gated using DAPI-stain before determining bacterial populations stained with CellROX-Green. Single stained cells were also used to set compensation parameters between dye signals. Statistical analysis was performed using the Mann–Whitney test on the Prism GraphPad Software 6.

### Growth phenotype of Hpx^–^ derivative strains

For growth studies, eight biological replicates of each strain were used (strains are listed in Supplemental File 1, Table S1). Cells were grown anaerobically in plates inside plastic bags using the BD GasPak EZ Pouch System (#260683-20) at 37°C for 3 days. Inoculation in 2 ml of freshly made anaerobic LB medium was performed inside anaerobic chamber BACTRONEZ (Bactron) filled with Anaerobic Mixed Gas (AMG—5% carbon dioxide, 5% hydrogen Balance Nitrogen Certified Standard Mixture—Airgas #X03NI90C2000024). The tubes were placed inside plastic bags using the BD GasPak EZ Pouch System (#260683-20) and incubated anaerobically overnight at 37°C, shaking at 200 rpm. The OD_600_ of overnight cultures were measured and normalized to 0.045–0.055 in 1 ml of LB. Still inside the chamber, the Bioscreen plate was prepared with 20 μl of each culture added by 180 μl of LB medium, resulting in a starting OD_600_ of 0.005. The plate was incubated aerobically in the Bioscreen system at 37°C, shaking continually, for 3 days. OD_600_ measurements were recorded every 30 min.

## Results

### Queuosine modification in tRNA affects *E. coli* sensitivity to metals

**Fig. 2:**
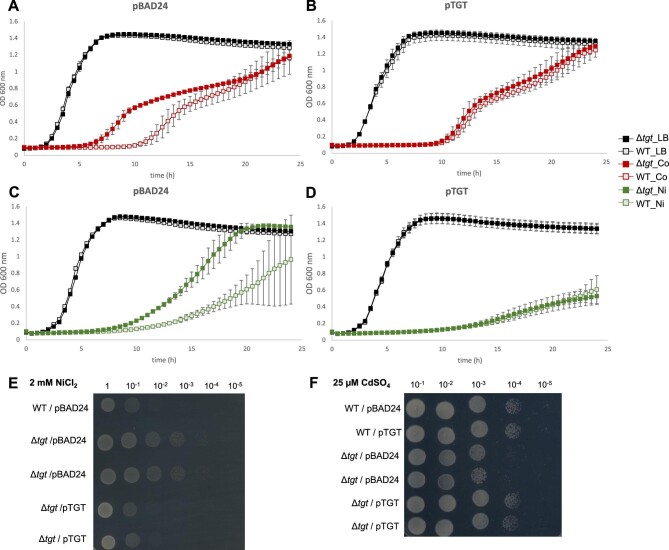
Metal-related phenotypes *of E. coli* Δ*tgt* strain. Growth of WT and Δ*tgt* strains was monitored in a Bioscreen C Analyzer at 37°C with constant shaking. Error bars showing standard deviation for biological triplicates. Both strains were transformed with pBAD24 (A and C) or pTGT (B and D). All growth experiments were performed in LB medium with 100 μg/ml ampicillin and 0.02% arabinose supplemented with 0.85 mM CoCl_2_ (A and B) or 2 mM NiCl_2_ (C and D). The same set of strains was tested for metal sensitivity assays on plates. For nickel sensitivity tests, overnight cultures in LB were diluted and grown to mid-exponential phase, then diluted to OD(A_600nm_) 1.0, and 7 μl of 10-fold serial dilutions were spotted on LB medium containing 2 mM NiCl_2_, 100 μg/ml ampicillin and 0.02% arabinose (E). For cadmium sensitivity tests, overnight cultures were diluted in low-phosphate (LP) medium containing ampicillin and grown to mid-exponential phase, then diluted to OD(A_600nm_) 1.0, and 7 μl of 10-fold serial dilutions were spotted on LP medium containing 25 μM CdSO_4_, 100 μg/ml ampicillin and 0.02% arabinose (F). Growth was analyzed after 24 h at 37°C. Spots corresponding to 10^–1^ to 10^–5^ dilutions are shown. WT, wild type; Ni, nickel; Co, cobalt.

Based on published observations linking Q or Q biosynthesis genes to metal metabolism,^30–34^ the role of Q under different metal stress conditions, such as excess Ni^2+^, Co^2+^, and Cd^2+^, was evaluated in *E. coli*. In LB medium, the *tgt* mutant, lacking Q in tRNA, showed higher resistance to Co^2+^ (0.85 mM) and to Ni^2+^ (2 mM) compared to the isogenic WT *E. coli* MG1655 strain (Fig. 2A and C). When following growth in microtiter plates (Bioscreen C), we found that, even though both WT and *tgt* mutant strains reach a similar final OD (A_600nm_) in stationary phase, the mutant showed a clear growth advantage. In the presence of Ni, for example, doubling times of 4 h for Δ*tgt* and of 5.3 h for WT were observed (Fig. 2A and C).

An independent systematic TnSeq fitness study^67^ (https://fit.genomics.lbl.gov) showed that insertions in the *tgt* gene of *E. coli* BW25113 strain provided growth advantage under nickel and cobalt stress (fitness value = 1.2). This means that the presence of this gene was detrimental for fitness in these conditions. In the same study, insertions in the *E. coli* genes *queA* and *queG* were also beneficial in these two metal stress conditions (Supplemental file 1, Table S3). This result is consistent with the nickel and cobalt resistance phenotype we observed in the *tgt* mutant.

The *tgt^–^* nickel resistance phenotype was also confirmed by plate assays (Fig. 2E). On the other hand, under cadmium stress (25 μM) in low phosphate minimal medium (MA), the *tgt* mutant was more sensitive than the WT strain (Fig. 2F). The three metal-related phenotypes were complemented by overexpressing the *tgt* gene in trans in pBAD24 (Fig. 2B, D, E, F), confirming that the absence of the *tgt* gene was responsible for the observed phenotype. Additional analyses showed that the nickel resistance phenotype of the ∆*tgt* mutant was caused by the absence of Q in tRNA, and not by the accumulation of Q precursors (preQ_0_ and preQ_1_) (Fig. S1, Results in Supplemental File 1).

### Transcriptomics analysis of wild-type strain exposed to high nickel

To understand the basis for the nickel resistance phenotype of the *tgt* mutant, we first analyzed the transcriptomic response of the WT strain to nickel stress to identify genes that were differentially expressed in nickel-treated WT compared to untreated WT (WT_Ni/WT_LB; Supplemental File 2). A total of 734 genes were found upregulated and 1642 genes downregulated in the treated cells. A similar analysis had already been performed^68^ but in different physiological conditions. In the previous study, cells were grown in M63 minimal medium and exposed to 50 μM NiCl_2_ for only 10 min, while in this study cells were grown in LB medium and exposed to 2 mM NiCl_2_ for 2 h, the same condition in which the *tgt* mutant phenotype of nickel resistance was observed.

In *E. coli*, as in other bacteria, nickel is used as a cofactor for enzymes such as Ni-Fe hydrogenases and glyoxalase I, but it is also toxic at high concentrations.^69,70^ The proposed causes of nickel toxicity involve the replacement of other essential metals in metalloproteins, the binding to catalytic residues of enzymes (or allosteric inhibition of catalytic site), and the generation of oxidative stress causing damage to DNA, proteins, or lipids.^70^ Therefore, well-controlled mechanisms for nickel import and distribution as well as for nickel efflux are required to avoid toxicity. Comparing the gene expression profiles allowed us to confirm that the main features of the nickel toxicity response were affected in our experiment

**Fig. 3:**
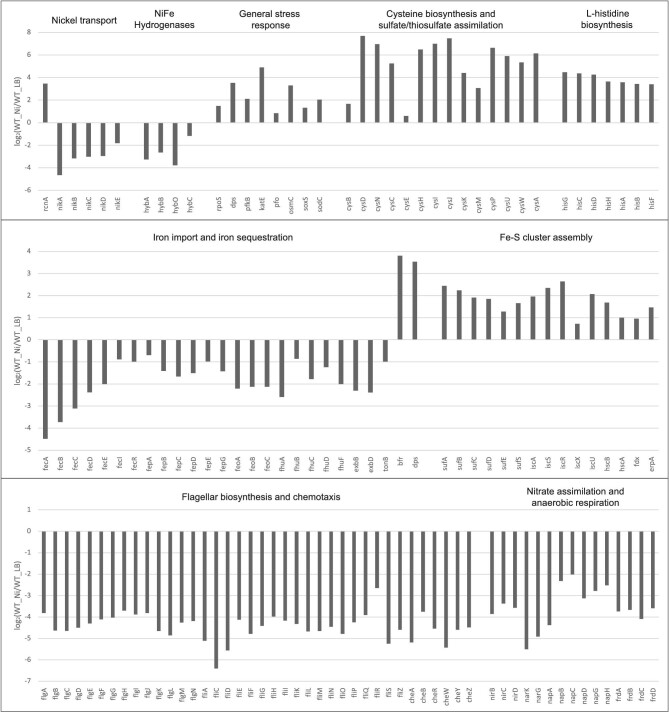
Transcripts differentially expressed in the wild-type strain upon exposure to excess nickel. Y-axis, differential gene expression reported as log_2_(FC) of mRNA levels in nickel-treated WT compared to untreated WT (WT_Ni/WT_LB). The genes were manually assigned to each category.

As expected upon exposure to excess nickel, genes involved in the general stress responses were induced in our and in the Gault *et al*. study, including the master stress regulator gene *rpoS*, and genes related to response to DNA damage and to oxidative stress, such as *dps, pfkB, katE, pfo, osmC, soxS*, and *sodC* (Fig. 3). While the overexpression of *rcnA*, the gene coding for the Ni and Co efflux pump was observed in both studies, the expected repression of the *nikABCDE* operon, which codes for the main Ni import system, was only observed in the current study (Fig. 3 and Supplemental file 2). One of the major findings of the Gault *et al*. study was that Ni stress activates systems involved in the defense against excess Fe and Cu, even if the contents of Fe and Cu remained unchanged. We did find that genes involved in iron import (*fec, fep, feo* and *fhu* genes, *exbBD, tonB*) were downregulated, while genes involved in iron sequestration (*bfr* and *dps*), as well as the Nickel-responsive Fe-uptake Regulator (*nfeR/yqjI*) were upregulated. Because we observed a strong response involving Fur (ferric uptake regulator) regulon, we compared our results to the study from McHugh *et al*.^71^ They analyzed the transcriptomics of *E. coli* under two conditions: iron starvation, by adding the iron chelator 2,2’-dipyridyl (DIP), and Fur absence, using a *fur* mutant. Whereas the majority of Fur regulated genes involved in iron acquisition were found upregulated during iron starvation and in the *fur* mutant, they were found downregulated in our study. This same effect was also observed by Gault *et al*. and suggests a nickel effect on Fur regulation.

Among the differences between our results and the Gault *et al*. study is the overexpression of genes coding for the universal stress proteins (*usp* genes) and the downregulation of *ryhB*, which we did not observe. Notably, genes encoding the two Fe-S cluster assembly systems, SUF and ISC, were overexpressed in the present study, but not in the Gault *et al*. study. Curiously, while genes involved in sulfate assimilation and cysteine biosynthetic process (*cys* genes) were found downregulated in the Gault *et al*. study, we found that the *cys* genes as well as genes involved in the histidine biosynthetic process (*his* genes) were among the most upregulated in our conditions (Fig. 3). It is known that nickel can bind His and Cys residues of enzymes catalytic sites,^70^ and it was observed that the addition of His and Cys was able to moderate the effects of Ni^2+^ on mouse Leydig cells *in vitro*.^72^ Thus, the increase in the synthesis of these two amino acids could be a protective reaction to nickel excess.

Among the repressed genes, there is a high prevalence of genes related to motility, mainly involved in flagellar biosynthesis and chemotaxis (*flg, fli, che* genes), and genes related to nitrate assimilation and anaerobic respiration (*nir, nar, nap, frd, hya, hyb, hyc* genes).

Regarding the Q biosynthesis pathway, Gault *et al.* detected a repression of *queA* and *queD* (*ygcM*), while we detected the repression of *queF* and *tgt*, which is consistent with the fitness data (Table S3 in Supplemental file 1) and with the nickel resistance phenotype of the *tgt* mutant (Fig. 2). To test if exposure to high nickel levels led to a reduction in levels of Q-modified tRNAs, we analyzed Q levels in tRNA^Asp^_GUC_ in the WT strain grown in 1.5 mM or 2.0 mM NiCl_2_ from one to three growth cycles using a Northern-type assay based on an acrylamide gel containing N-acryloyl-3-aminophenylboronic acid (APB), which slows migration of Q-containing tRNA.^11,73^ Under the tested conditions, the presence of high nickel did not affect Q levels in tRNA^Asp^ (Fig. S2 in Supplemental file 1).

### Transcriptomics of the *tgt* mutant: stress, protein stability, cell respiration, and metal import are the main biological processes affected by the absence of Q

After looking at changes in *E. coli* gene expression patterns upon exposure to nickel stress, we focused on analyzing the impact of the absence of *tgt* and, consequently, of Q in tRNA. Using RNA-seq, we compared mRNA levels in the *tgt* mutant and in the WT strain (*E. coli* MG1655). Differential gene expression is reported as log_2_(FC) of mRNA levels in the *tgt* mutant compared to the WT strain (tgt_LB/WT_LB) (Supplemental File 2). In this condition, 417 genes were found upregulated and 1053 genes downregulated in the *tgt* mutant.

GO terms enrichment was performed using DAVID Bioinformatics Resources 6.8^74^ for differentially expressed genes in the comparisons of the *tgt* mutant to the WT strain in LB medium (tgt_LB/WT_LB), and of nickel-treated and untreated WT (WT_Ni/WT_LB). The complete list of enriched biological processes and their respective genes can be accessed in Supplemental file 3. The most prominent observed biological processes in the *tgt* mutant were manually combined in broader categories and displayed in Table 1, where some of the genes illustrate each category.

**Table 1.** Genes with altered expression in the *tgt* mutant

Gene name	Product	log_2_(tgt_LB/WT_LB)
Main upregulated biological processes		
*Stress-induced genes*		
ibpA	Small heat shock protein IbpA	2.74
ibpB	Small heat shock protein IbpB	2.92
clpP	Serine protease	0.65
clpX	ClpX ATP-dependent protease specificity component and chaperone	0.74
dnaK	Chaperone protein DnaK	1.27
dnaJ	Chaperone protein DnaJ	1.23
grpE	Nucleotide exchange factor in the DnaK-DnaJ-GrpE chaperone system	0.99
Lon	Lon protease	1.56
ldhA	D-Lactate dehydrogenase—fermentative	0.91
mutM	Formamidopyrimidine DNA glycosylase	2.36
hslR	Heat shock protein Hsp15	1.15
hslU	ATPase component of the HslVU protease	1.58
hslV	Peptidase component of the HslVU protease	2.09
hslO	Molecular chaperone Hsp33	1.53
hspQ	Heat shock protein, hemimethylated DNA-binding protein	1.09
hflC	Regulator of FtsH protease	0.52
hflK	Regulator of FtsH protease	0.74
hflX	Ribosome-dissociating factor, GTPase	0.62
ybeY	Endoribonuclease	0.67
*Aerobic respiration and TCA cycle*		
cyoA	Cytochrome bo3 terminal oxidase subunit II	1.15
cyoB	Cytochrome bo3 terminal oxidase subunit I	0.99
cyoC	Cytochrome bo3 terminal oxidase subunit III	0.87
cyoD	Cytochrome bo3 terminal oxidase subunit IV	0.88
erpA	Essential respiratory protein A	1.44
glpD	Glycerol-3-phosphate dehydrogenase, aerobic	0.57
nuoF	NADH:ubiquinone oxidoreductase, chain F	0.69
acnB	Bifunctional aconitate hydratase 2 and 2-methylisocitrate dehydratase	0.69
aceB	Malate synthase A	1.10
sucA	2-Oxoglutarate decarboxylase, thiamine-requiring	0.77
nlpD	Murein hydrolase activator	0.88
sdhA	Succinate:quinone oxidoreductase, FAD binding protein	1.27
sdhB	Succinate:quinone oxidoreductase, iron-sulfur cluster binding protein	0.78
sdhC	Succinate:quinone oxidoreductase, membrane protein	1.27
sdhD	Succinate:quinone oxidoreductase, membrane protein	1.66
fumA	Fumarase A	1.20
fumC	Fumarase C	1.17
Mqo	Malate:quinone oxidoreductase	1.95
*Fatty-acid biosynthetic and oxidation processes*		
fabA	Beta-hydroxyacyl-ACP dehydratase/isomerase	0.69
fabB	Beta-ketoacyl-ACP synthase I	1.19
fabF	Beta-ketoacyl-ACP synthase II	0.66
fabH	Beta-ketoacyl-ACP synthase III	0.75
fadA	3-Ketoacyl-CoA thiolase	1.26
fadB	Fatty acid oxidation complex, α component	2.18
fadD	Fatty acyl-CoA synthetase	1.49
fadJ	FadJ component of anaerobic fatty acid oxidation complex	1.00
accA	Acetyl-CoA carboxyltransferase subunit α	0.66
accC	Biotin carboxylase	0.66
*Protein metabolism (translation fidelity, stability, folding, regulation and degradation)*		
miaA	tRNA(i6A37) synthase	1.02
tsaC	Threonylcarbamoyl-AMP synthase	0.61
Dtd	D-Tyr-tRNATyr deacylase	0.86
tilS	tRNAIle-lysidine synthetase	0.61
slyD	FKBP-type peptidyl prolyl cis-trans isomerase	0.63
ftsH	ATP-dependent zinc metalloprotease FtsH	0.92
htpX	Heat shock protein, protease	1.17
sohB	S49 peptidase family protein	0.81
pepN	Aminopeptidase N	0.78
*Fe-S cluster assembly (ISC system and associated genes)*		
iscA	Iron-sulfur cluster assembly protein	1.63
iscS	Cysteine desulfurase	2.07
iscR	IscR DNA-binding transcriptional dual regulator	2.19
iscX	Regulator of iron-sulfur cluster assembly	1.03
iscU	Scaffold protein for iron-sulfur cluster assembly	1.75
hscB	Co-chaperone for [Fe-S] cluster biosynthesis	1.85
hscA	Chaperone for [Fe-S] cluster biosynthesis	1.32
Fdx	Oxidized ferredoxin/reduced ferredoxin	1.04
cyaY	Frataxin CyaY	0.41
erpA	Essential respiratory protein A	1.44
nfuA	Iron-sulfur cluster scaffold protein	1.50
*Amino acid biosynthetic pathway*		
hisA	Phosphoribosylformimino-5-aminoimidazole carboxamide ribotide isomerase	1.41
hisB	Imidazoleglycerol-phosphate dehydratase/histidinol-phosphatase	1.00
hisC	Histidinol-phosphate aminotransferase	1.20
hisD	Histidinal dehydrogenase/histidinol dehydrogenase	1.09
hisF	Imidazole glycerol phosphate synthase, HisF subunit	1.42
hisG	ATP phosphoribosyltransferase	1.29
hisH	Imidazole glycerol phosphate synthase, HisH subunit	1.26
hisI	Histidine biosynthesis bifunctional protein HisIE	1.16
aroA	3-Phosphoshikimate 1-carboxyvinyltransferase	0.54
aroB	3-Dehydroquinate synthase	0.34
aroC	Chorismate synthase	0.51
aroE	Shikimate dehydrogenase	1.17
aroF	2-Dehydro-3-deoxyphosphoheptonate aldolase	0.45
trpA	Tryptophan synthase, alpha subunit	0.75
trpB	Tryptophan synthase, beta subunit	1.08
trpC	Fused indole-3-glycerol phosphate synthase	1.29
Asd	Aspartate semialdehyde dehydrogenase	0.77
thrA	Aspartate kinase/homoserine dehydrogenase	0.57
metL	Aspartate kinase/homoserine dehydrogenase	0.42
dapB	4-Hydroxy-tetrahydrodipicolinate reductase	0.55
dapF	Diaminopimelate epimerase	1.02
Main down regulated biological processes		
*Anaerobic respiration and fermentation*		
dmsA	Dimethyl sulfoxide reductase, chain A	–3.07
dmsB	Dimethyl sulfoxide reductase, chain B	–2.07
dmsC	Dimethyl sulfoxide reductase, chain C	–1.64
fdnG	Formate dehydrogenase N subunit α	–2.32
frdA	Fumarate reductase flavoprotein	–0.76
frdB	Fumarate reductase iron-sulfur protein	–0.85
frdC	Fumarate reductase membrane protein	–0.75
frdD	Fumarate reductase membrane protein	–0.77
hyaA	Hydrogenase 1, small subunit	–0.58
hyaB	Hydrogenase 1, large subunit	–0.52
hyaC	Hydrogenase 1, b-type cytochrome subunit	–0.82
hybA	Hydrogenase 2, [Fe-S] binding, ferredoxin-type component HybA	–1.69
hybB	Hydrogenase 2, integral membrane subunit HybB	–1.14
hybO	Hydrogenase 2, small subunit	–2.36
hycB	Hydrogenase 3, Fe-S subunit	–0.51
napA	Large subunit of periplasmic nitrate reductase, molybdoprotein	–2.68
napB	Periplasmic nitrate reductase cytochrome c550 protein	–1.09
napC	Periplasmic nitrate reductase, cytochrome c protein	–1.62
narG	Nitrate reductase A subunit α	–4.28
narH	Nitrate reductase A subunit β	–2.43
nirB	Nitrite reductase, large subunit	–4.00
nirD	Nitrite reductase, small subunit	–3.66
*Metal import, incorporation, and homeostasis*		
nikA	Nickel ABC transporter—periplasmic binding protein	–3.61
nikB	Nickel ABC transporter—membrane subunit	–2.83
nikC	Nickel ABC transporter—membrane subunit	–2.77
nikD	Nickel ABC transporter—ATP binding subunit	–2.30
nikE	Nickel ABC transporter—ATP binding subunit	–1.73
hypA	Accessory protein for nickel incorporation into hydrogenase 3	–2.84
hypB	Accessory protein for nickel incorporation into hydrogenase isoenzymes	–3.00
hypD	Scaffold protein for assembly of the Fe-(CN)2CO cofactor	–1.89
feoA	Ferrous iron transport protein A	–3.03
feoB	Ferrous iron transporter FeoB	–3.00
feoC	Ferrous iron transporter FeoC	–2.76
fepA	Ferric enterobactin/colicin B/colicin D outer membrane porin FepA	–0.67
fepB	Ferric enterobactin ABC transporter—periplasmic binding protein	–0.67
fepC	Ferric enterobactin ABC transporter—ATP binding subunit	–1.09
fepE	Ferric enterobactin (enterochelin) transport	–0.82
fepG	Ferric enterobactin ABC transporter—membrane subunit	–0.98
fhuA	Ferrichrome outer membrane transporter/phage receptor	–1.62
fhuC	Iron (III) hydroxamate ABC transporter—ATP binding subunit	–1.28
fhuD	Iron (III) hydroxamate ABC transporter—periplasmic binding protein	–0.91
fhuE	Ferric coprogen outer membrane porin FhuE	–0.51
entE	Enterobactin synthetase component E	–0.95
entD	Enterobactin synthetase component D	–0.66
cirA	Ferric dihyroxybenzoylserine outer membrane transporter	–0.64
tonB	TonB energy transducing system—TonB subunit	–0.64
exbB	TonB energy transducing system—ExbB subunit	–1.56
exbD	TonB energy transducing system—ExbD subunit	–1.87
Dps	Stationary phase nucleoid protein that sequesters iron and protects DNA from damage	–0.81
yqjH	Ni-responsive Fe-uptake flavoprotein (nfeF)	–0.77
ftnB	Predicted ferritin-like protein	–0.71
Fes	Enterochelin esterase	–0.98
ryhB	RyhB small regulatory RNA involved in iron homeostasis	–1.87
*Fe-S cluster assembly (Suf system)*		
sufA*	Fe-S transport protein in Fe-S cluster assembly	–0.50
sufB*	SufB component of SufBCD Fe-S cluster scaffold complex	–0.55
sufC	SufC component of SufBCD Fe-S cluster scaffold complex	–0.71
sufD	SufD component of SufBCD Fe-S cluster scaffold complex	–0.84
sufE	Sulfur acceptor for SufS cysteine desulfurase	–0.96
sufS	L-Cysteine desulfurase	–0.80

Transcriptomics of the ∆*tgt* compared to WT strain in LB medium. The most affected biological processes were manually combined in broader categories, with some genes illustrating each category. The complete list of enriched biological processes and their respective genes can be accessed in Supplemental file 3. *, genes with negative ratios but not considered significantly altered.

Genes involved in aerobic respiration, TCA cycle, and fatty-acid biosynthetic and oxidation processes are found upregulated in the *tgt* mutant in comparison to the WT strain (Table 1). Conversely, genes involved in anaerobic respiration and fermentation are downregulated. There is an increase in expression of the entire ISC operon, the housekeeping system for iron-sulfur cluster [Fe-S] assembly, while there is a decrease in expression of most of the SUF operon genes, responsible for [Fe-S] assembly in stress situations (such as oxidative stress and low iron) (Table 1). A massive number of genes involved in metal import, mainly of iron, cobalt, and nickel, are repressed. The changes in the nickel homeostasis response genes are discussed in more details below. The increased expression of many stress-induced genes suggests a response to misfolded proteins and DNA damage being triggered in the absence of Q modification in tRNA (Table 1). It is also observed a striking number of upregulated genes belonging to amino acid biosynthesis pathways (mainly Trp, Phe, Tyr, His, Ala, Lys, and Met) and processes related to protein translational fidelity, protein stability and folding, and catabolism of misfolded proteins. This regulation points to a role of Q in protein translation and homeostasis. Other biological processes observed with differentially expressed genes in the *tgt* mutant are the upregulated cell shape and division, putrescine catabolism, and glycine betaine biosynthesis. Among the downregulated processes there are also biofilm formation and cell adhesion, flagellum synthesis, DNA recombination and transposition, pilus organization, ethanolamine catabolism, lipopolysaccharide biosynthesis, and carnitine and pyrimidine metabolic process (Table 1 and Supplemental file 3).

### Proteomics of the *tgt* mutant confirms altered biological processes identified in the transcriptomics analysis

Total extracts of *E. coli* WT and *tgt* mutant strains in LB medium, prepared from the same cultures used for the RNA-seq experiment, were labeled with TMT Isobaric Mass Tags and analyzed by chromatography-coupled Orbitrap mass spectrometry as described in Methods section. This analysis produced 23 326 spectra that were assigned to 855 *E. coli* proteins (peptide threshold: <1% FDR) across all three experimental replicates (protein threshold: >99% confidence, minimum of 2 peptides). Fold-change values were calculated to obtain relative protein abundance (*tgt*_LB/WT_LB). Proteomics data is available in Supplemental File 4 and was uploaded to the CHORUS database (https://chorusproject.org/anonymous/download/experiment/2a0b77c87014485a993df01e7744a5a6) under ID 3655.

In the *tgt* mutant, 37 proteins were found to be highly upregulated, and 33 downregulated when compared to WT (Table 2). The QueD protein, an enzyme that participates in the synthesis of Q precursors,^6^ is the most abundant protein in the mutant, suggesting a possible feedback regulation caused by the absence of Q in tRNA. To date, no information about the regulation of the Q biosynthetic pathway is known for *E. coli*, and this could be the first time such a feedback mechanism is observed. Among the most abundant proteins, DdlA participates in the peptidoglycan biosynthesis, and the *ddlA* mutant was shown to be 8-fold more sensitive to X-ray radiation.^75^ The phosphohistidine phosphatase SixA dephosphorylates the His717 of ArcB,^76^ whose protein levels are also increased in the *tgt* mutant. Another interesting protein with increased levels in the mutant is the putative ATP-dependent protease YcbZ, which is linked to translation. The *ycbZ* mutant showed increased levels of stop codon readthrough, and the use of a complementation plasmid rescued the fidelity and fitness defects of the mutant.^77^ The least abundant protein in the mutant is YoaE. This member of the UPF0053 is not functionally characterized but GEO profiles searches shows that *yoaE* expression is induced in the *fur* mutant and repressed in the WT when these strains are exposed to gentamicin (Profile GDS5162/1768340_s_at, https://www.ncbi.nlm.nih.gov/geoprofiles/). *yoaE* expression is also induced in a strain deficient for the hydrogen peroxide-scavenging enzymes (Profile GDS5163/1768340_s_at).

**Table 2.** Proteins with increased or decreased abundance in the *tgt* strain compared to the WT strain in LB medium

Gene name	Product	log_2_(*tgt*_LB/WT_LB)
*Proteins with increased abundance in the tgt mutant*		
queD	6-Carboxy-5,6,7,8-tetrahydropterin synthase	1.15
ddlA	D-Alanine—D-alanine ligase A	1.13
sixA	Phosphohistidine phosphatase SixA	1.09
ycbZ	Putative ATP-dependent protease YcbZ	1.06
fadE	Acyl-CoA dehydrogenase	1.00
gspD	Type II secretion system protein GspD	1.00
yhjJ	Peptidase M16 family protein YhjJ	0.98
yidZ	Putative LysR-type transcriptional regulator YidZ	0.94
cyoA	Cytochrome bo3 ubiquinol oxidase subunit 2	0.84
rluC	23S rRNA pseudouridine synthase C	0.83
sbcB	Exodeoxyribonuclease I	0.81
yeiG	S-Formylglutathione hydrolase/S-lactoylglutathione hydrolase	0.81
ecpA	Common pilus major subunit	0.78
polB	DNA polymerase II	0.77
yjbI	Pentapeptide repeats-containing protein YjbI	0.76
tynA	Copper-containing amine oxidase	0.75
ldhA	D-Lactate dehydrogenase	0.73
tdcA	DNA-binding transcriptional activator TdcA	0.73
miaB	Isopentenyl-adenosine A37 tRNA methylthiolase	0.73
alkB	DNA oxidative demethylase	0.72
gcvT	Aminomethyltransferase	0.71
hemB	Porphobilinogen synthase	0.70
Tsr	Serine chemoreceptor protein	0.68
yeiP	Elongation factor P family protein	0.68
parC	Dimer of DNA topoisomerase IV subunit A	0.68
gshA	Glutamate—cysteine ligase	0.68
Lrp	Leucine-responsive regulatory protein	0.67
yjiY	Pyruvate/proton symporter BtsT	0.67
yfgI	Nalidixic acid resistance protein YfgI	0.64
casC	CRISPR system Cascade subunit CasC	0.64
ybaQ	DNA-binding transcriptional regulator YbaQ	0.63
waaH	UDP-glucuronate:LPS(HepIII) glycosyltransferase	0.62
yfcE	Phosphodiesterase YfcE	0.62
arcB	Aerobic respiration control sensor protein ArcB	0.60
yfcD	Putative Nudix hydrolase YfcD	0.60
nuoB	NADH:quinone oxidoreductase subunit B	
*Proteins with decreased abundance in the tgt mutant*		
yoaE	Putative inner membrane protein	–1.87
seqA	Negative modulator of initiation of replication	–1.15
allD	Ureidoglycolate dehydrogenase	–1.11
mutM	Formamidopyrimidine-DNA glycosylase	–1.07
livM	Branched chain amino acid/phenylalanine ABC transporter membrane subunit LivM	–1.02
Wzc	Protein-tyrosine kinase Wzc	–0.99
cobS	Cobalamin 5'-phosphate synthase	–0.98
mrcA	Penicillin-binding protein 1A PBP1A	–0.93
torC	Cytochrome c menaquinol dehydrogenase TorC	–0.87
wecB	UDP-N-acetylglucosamine 2-epimerase	–0.86
rbsK	Ribokinase	–0.84
fhuA	Ferrichrome outer membrane transporter/phage receptor	–0.81
xylE	D-Xylose/proton symporter	–0.81
sgbH	3-Keto-L-gulonate-6-phosphate decarboxylase SgbH	–0.79
mhpA	3-(3-Hydroxy-phenyl)propionate/3-hydroxycinnamic acid hydroxylase	–0.76
fhuB	Iron(III) hydroxamate ABC transporter membrane subunit	–0.75
rhsA	rhs element protein RhsA	–0.75
rcsB	DNA-binding transcriptional activator RcsB	–0.74
ihfB	Integration host factor subunit beta	–0.73
ascB	6-Phospho-beta-glucosidase AscB	–0.69
yigZ	IMPACT family member YigZ	–0.68
fliM	Flagellar motor switch protein FliM	–0.67
nikB	Nickel transport system permease protein NikB	–0.67
srlQ	D-Arabinose 5-phosphate isomerase GutQ	–0.66
ycaQ	Winged helix DNA-binding domain-containing protein YcaQ	–0.65
frvA	Putative PTS enzyme IIA component FrvA	–0.64
bioH	Pimeloyl-acyl carrier protein methyl ester esterase	–0.63
Map	Methionine aminopeptidase	–0.61
entF	Enterobactin synthase component F	–0.60
ptrA	Protease 3	–0.60
otsA	Trehalose-6-phosphate synthase	–0.60
glyQ	Glycine-tRNA ligase alpha subunit	–0.59
yjeJ	Uncharacterized protein YjeJ	–0.59

Proteins with variable abundance were considered for log_2_(*tgt*_LB/WT_LB) higher than 0.59 and lower than –0.59.

A comparison between gene expression (transcriptomics) and protein abundance (proteomics) is displayed in Supplemental File 5. As expected,^78^ we found a poor correlation between mRNA and protein levels, even using the same batch of cell cultures for both analyses. This difference is likely due to the different roles of transcription and translation in regulating the levels of individual proteins under these specific conditions,^79^ with a potential role for translational regulation by the tRNA epitranscriptome, including Q modification.^80–82^

Examination of the concordant increases or decreases in mRNA levels and protein abundances can give insights into the regulators responsible for disturbances of biological processes observed in the *tgt* mutant (Table 3). The upregulation of both mRNA and protein levels of acyl-CoA dehydrogenase (*fadE*) and cytochrome *bo_3_* ubiquinol oxidase subunit 2 (*cyoA*) confirms the induction of the fatty-acid oxidation and aerobic processes in the mutant. A feature shared by *fadE, cyoA*, and *ldhA* is their repression by the transcriptional dual regulator ArcA. The derepression of these genes suggests that ArcA is not active in the *tgt* mutant. Concordant downregulation is observed for nickel (*nikB*) and iron import (*entF, fhuA*) related genes. The latter two are repressed by Fur, while *nikB* and *entF* are both induced by FNR, suggesting that Fur is active in the *tgt* mutant and FNR is not. The other genes with concordant decrease in mRNA and protein levels are controlled by different regulators and sigma factors.

**Table 3.** Concordant and discordant variations of mRNA and protein levels in the *E. coli tgt* mutant in LB medium

Gene name	Transcriptomics	Proteomics
	Concordant variations	
	Increased mRNA levels	Increased protein levels
fadE	0.79	1.00
cyoA	1.15	0.84
ldhA	0.91	0.73
	Decreased mRNA levels	Decreased protein levels
otsA	–0.75	–0.60
entF	–1.21	–0.60
nikB	–2.82	–0.67
rhsA	–0.58	–0.75
sgbH	–0.92	–0.79
fhuA	–1.62	–0.81
Wzc	–0.67	–0.99
	Discordant variations	
	Decreased mRNA levels	Increased protein levels
tdcA	–3.38	0.73
gspD	–0.65	1.00
	Increased mRNA levels	Decreased protein levels
mutM	2.36	–1.07
rbsK	0.56	–0.84

The numbers reflect the log_2_(*tgt*_LB/WT_LB).

On the other hand, some genes display very discordant variations between mRNA and protein levels. For example, the type II secretion system protein GspD, whose gene is repressed by Fur, and the transcriptional activator TdcA, activated by FNR, show downregulation of mRNA levels but increased protein levels in the absence of *tgt*. Inversely, the ribokinase RbsK and the DNA glycosylase MutM, controlled by sigma factors 70 and 32, respectively, show increased mRNA levels but decreased protein abundance (Table 3).

### Investigation of Q influence on codon usage

Given the role of wobble Q in modulating reading of NAU and NAC synonymous codons by G_34_U_35_N_36_ anticodons on tRNAs corresponding to Asn, Asp, His, and Tyr,^1^ we assessed the codon usage patterns using our transcriptomics and proteomics data.

Exploring our transcriptomics dataset, we analyzed the differences in the relative frequencies of the codons NAC and NAU (of the amino acids Tyr, His, Asp, and Asn). We observed that the frequencies of these codons are not consistently elevated or depleted in the genes with differential expression when compared with their genomic frequencies (data not shown).

Using proteomics fold-change data for proteins upregulated and downregulated by the loss of Q-inserting *tgt* (Supplemental File 4), we calculated codon usage frequency relative to genome average for the top 37 up- and 33 down-regulated proteins, and then analyzed this data relative to protein up- and down-regulation values by principal components analysis. We found only a weak discrimination of the protein changes based on codon usage in the scores plot, although there was a strong separation of NAC and NAU codons that represent targets for Q-modified tRNAs (Fig. S3 in Supplemental file 1). This suggests that there is a modest influence of the loss of Tgt enzyme and Q modification on translation of proteins encoded by genes enriched with Q-dependent codons, with a similar influence of other codons and other factors.

### The nickel resistance phenotype of the *tgt* mutant is caused by downregulation of the nickel import system NikABCDE

**Fig. 4:**
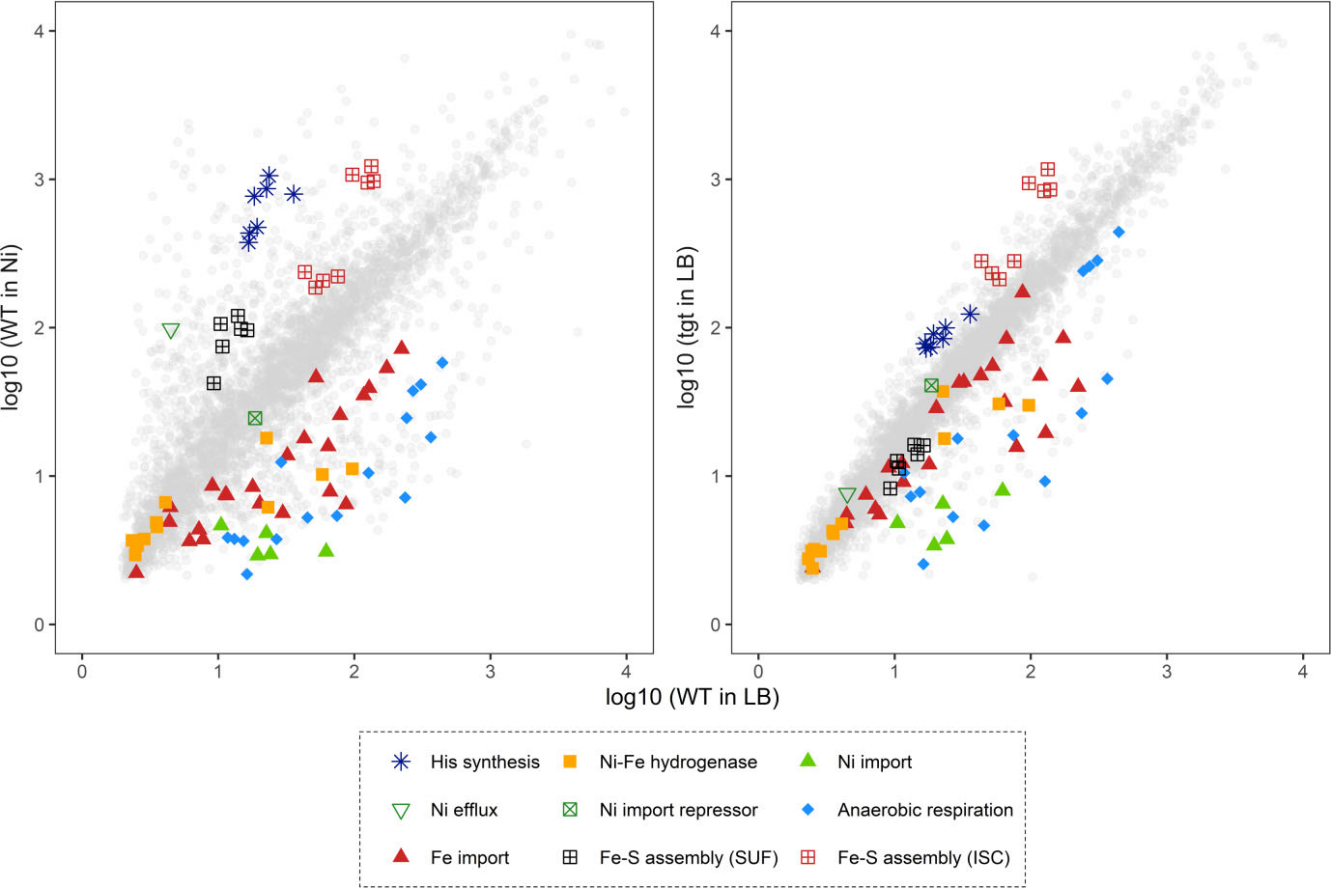
Gene expression profiles of the nickel-treated WT strain and the untreated *tgt* mutant. Expression levels of genes in nickel-treated WT (graph on the left) and genes in untreated *tgt* mutant (graph on the right) are plotted against expression levels of genes in untreated WT. Genes from some enriched biological processes are highlighted with different symbols and colors. Supplemental file 6 contains the list of the plotted genes and expression values.

One of the most striking observations about the perturbation in gene expression caused by the absence of queuosine modification (*tgt*_LB/WT_LB) was the strong overlap with the response observed for the WT cell exposed to nickel (WT_Ni/WT_LB). As highlighted in Fig. 4, similar induction of the Fe-S cluster assembly ISC system, of the histidine biosynthesis pathway, and of amino acid and protein biosynthesis and transport was observed. Conversely, in both conditions, genes involved in anaerobic respiration and in metal import were strongly repressed. The downregulation of the entire *nikABCDE* operon, which codes for the main nickel import system in the cell, could explain the observed nickel resistance phenotype of the *tgt* mutant. The downregulation of *nikB* was observed both at transcriptional and translational levels, in our RNAseq and proteomics analyses (Tables 1–3).

**Fig. 5:**
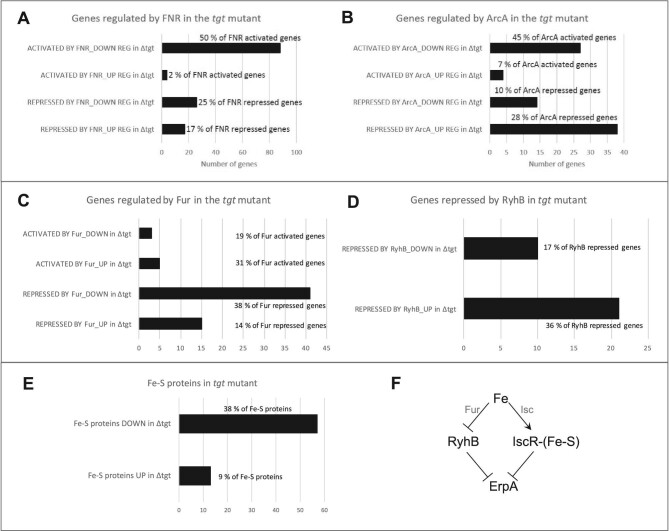
Regulons expression in the *tgt* mutant. (A) Genes regulated by FNR; (B) genes regulated by ArcA; (C) genes regulated by Fur; (D) genes repressed by RyhB. Graphs show the number of genes (X axis) activated and repressed by each major regulator and their respective regulation in the *tgt* mutant (upregulated or downregulated based on mRNA levels). To the right of each bar, the percentage represented by the number of genes among the total activated or repressed genes by each regulator. (E) number of Fe-S proteins (X axis) whose mRNA levels are upregulated or downregulated in the *tgt* mutant compared to the WT strain in LB medium. The percentages represented by these numbers among the total Fe-S proteins in *E. coli* were included in the graph. (F) Schematic representation of the incoherent regulation by Fur and Isc proteins. In the presence of abundant Fe levels, Fur represses *ryhB*, thus derepressing the expression of *erpA*; while IscR bound to Fe-S cluster (holo-IscR) represses *erpA*.^91^

The repression of the *nikABCDE* operon along with genes for Ni-Fe hydrogenases (*hya/hyb/hyc* genes) and nitrite/nitrate reductases (*nir/nar* genes) indicate a possible reduction in the activity of the FNR regulator. Because, in the past, a *tgt* mutant strain was found deleted for the *fnr* gene,^83^ we checked and confirmed that the *tgt* mutant strain we used in our RNAseq transcriptomics contains the *fnr* gene and it is not mutated (confirmed by checking the sequence of *fnr* transcripts in the transcriptomics data, and by sequencing of PCR products of the *fnr* gene amplified from the WT and *tgt* mutant strains—data not shown). We also confirmed that *fnr* is expressed at transcriptional (observed in the transcriptomics data) and translational levels (detected by Western blot—Fig. S4). Besides the altered expression of the FNR regulon, the activity of another major regulator of anaerobic response, ArcA, seems to be disturbed in the cell. The expression levels of genes regulated by FNR and ArcA indeed point to a repression of the activity of these two major regulators (Fig. 5A and B), leading therefore to a repression of the anaerobic metabolism.

### Expression of the iron import system is repressed in the Q^–^ strain

While FNR and ArcA activities seem to be repressed, an induction of the activity of the master regulator Fur is clear in the *tgt* mutant. Most of the *E. coli* iron homeostasis genes belong to the Fur regulon [dataset] (https://regulondb.ccg.unam.mx/)^84^ and many of these genes are repressed in the *tgt* mutant (Fig. 5C, Table 1). This includes genes involved in iron import and synthesis of siderophores, such as *feoABC, fepABCEG, fhuACDE, nfeF* (*yqjH*), *entED, cirA*, and *tonB/exbBD*. Consistently with most of Fur regulon genes, downregulation of *ryhB* expression was observed in the *tgt* mutant. RyhB is a small RNA that represses the expression of iron-containing proteins, hence reducing iron consumption under low-iron conditions.^85^ In accordance with the Q^–^ strain transcriptomics data, repression of *ryhB* and consequent derepression of its regulon corroborates the induction of *sodB*, of genes in the TCA cycle and aerobic respiratory chain, and of the ISC operon (Fig. 5D, Table 1). Because Fur is activated by Fe^2+^,^86^ the observed expression profiles could be a result of overexpression of Fur and/or increased intracellular iron levels. However, analyses reported in the Supplemental sections showed that no differences in *fur* expression or FUR protein levels were observed (Supplemental file 2 and Fig. S5A, in Supplemental file 1), as well as no difference in *ryhB* promoter activity (Fig. S5B). And no significant difference was detected in free iron levels between the WT and the *tgt* mutant when measured by EPR (Fig. S5D), even if a slight sensitivity of the mutant to the iron activated antibiotic streptonigrin^87^ was observed (Fig. S5C).

### Absence of Q in tRNA perturbs the expression of Fe-S cluster assembly systems

Although the EPR result has not confirmed an increased amount of free iron in the *tgt* mutant, the increased sensitivity to streptonigrin (Fig. S5C in Supplemental file 1) and the repression of iron import genes, concurrently with an induction of the *isc* operon (*iscRSUA-hscBAfdx*) (Table 1), pointed to an imbalance in levels of Fe-S clusters in the *tgt* mutant. The DNA-binding transcriptional dual regulator IscR is sensitive to changes in the Fe-S pools during aerobic conditions. When bound to a [2Fe-2S] cluster, holo-IscR represses the *isc* operon by directly binding to the *iscR* promoter. When not bound to the cluster, apo-IscR cannot bind to the *iscR* promoter; thus, repression is alleviated and the *isc* operon is expressed—reviewed in.^88,89^ Apo-IscR, instead, can bind to the promoter of the *suf* operon and activate its expression. Besides the Fe-S pools, Fe levels and oxidative stress can also influence the regulation between these two Fe-S cluster assembly systems. Under iron-replete conditions, Fur directly represses the expression of the *suf* operon, and indirectly induces the *isc* operon through repression of *ryhB* expression, consequently preventing RyhB to degrade the *iscSUA* mRNA. Oxidative stress participates in this regulation by possibly causing damage to the Fe-S cluster bound to IscR, and by OxyR induction of the *suf* operon.^88^ Fitting the transcriptomics data of the *tgt* mutant in this regulation network suggests that, in the absence of Q, the *suf* operon and *ryhB* are repressed by Fur-Fe^2+^, while the *isc* operon is derepressed possibly because of the formation of apo-IscR and the absence of RyhB. Another hint that IscR is in its apo form is the upregulation of *erpA* in the mutant (log_2_FC + 1.44). The gene *erpA* codes for an A-type carrier that delivers Fe-S clusters to apoproteins.^90^ Its expression is repressed by both RyhB and holo-IscR. However, these two repressors act in opposite conditions regarding Fe levels, which is considered an incoherent regulation.^91^ Under Fe abundant condition, *ryhB* is repressed, alleviating *erpA* expression. On the other hand, the formation of holo-IscR is favored, which keeps *erpA* repressed. However, if IscR is in its apo form, then *erpA* expression will be alleviated from the two repressors (see scheme in Fig. 5F). Mandin *et al*. proposed that this mechanism of regulation enables *erpA* expression only when it is most needed for Fe-S protein maturation.^91^ In the *tgt* mutant, most of RyhB repressed genes are upregulated, including *erpA*, suggesting apo-IscR is present. Curiously, the ErpA protein level is slightly reduced in the *tgt* mutant compared to the WT level (log_2_FC -0.51). Regarding Fe-S proteins, a high percentage of these proteins are downregulated in the *tgt* mutant (Table S4 in Supplemental File 1). Although RyhB controls the expression of several Fe-S proteins, and a high percentage of the RyhB regulon is upregulated (Fig. 5D), the expression of a great proportion of the Fe-S proteins is repressed (Fig. 5E, Table S4). This regulation pattern suggests that the *tgt* mutant is inducing the assembly of Fe-S clusters by the *isc* operon at the same time that it is restricting the consumption of the clusters by Fe-S proteins. Another regulation pointing to the favored formation of apo-IscR in the *tgt* mutant is the enrichment of genes for biofilm formation among the mutant repressed genes (Supplemental File 3). A decrease in levels of Fe-S clusters, due to environmental stresses or genetic defects in cluster assembly, is thought to inhibit biofilm formation and induce bacterial dispersal to a more favorable environment.^92^ To test if there was an imbalance in Fe-S cluster levels in the *tgt* mutant, we measured the activities of promoters regulated by Fe-S proteins, but found no difference in their expression in the absence of Q in tRNA (Fig. S6, see also methods and results in Supplemental file 1).

### Transcriptional regulation in the *tgt* mutant mimics the transition to increased O_2_ levels

Since the differences in Fe and Fe-S levels did not seem to be the cause of the regulation observed in the *tgt* mutant, we decided to investigate the influence of oxygen. We compared the expression profiles of the *tgt* mutant to two studies that analyzed the dynamic transcriptome of *E. coli* K12 MG1655 during transitions to higher or lower O_2_ levels.^93,94^ One important difference between these studies is that Partridge *et al*. grew the cells in Evans-defined medium supplemented with glucose while we used LB medium. Although the transition from aerobic to micro-aerobic conditions^94^ is not an exact reversal of the anaerobic to aerobic transition^93^ and not precisely comparable to the conditions in the current study, some informative comparisons could be made (Table 4).

**Table 4.** Comparison between the transcriptomics of the *tgt* mutant of *E. coli* responses to transitions to reduced and increased oxygen levels (Partridge et al., 2006, 2007)

	Aerobic to microaerobic (reduced O_2_ levels) (Partridge et al., 2007)	Anaerobic to aerobic (increased O_2_ levels) (Partridge et al., 2006)	*tgt* mutant in aerobic conditions (this study)
**Central metabolism**			
FNR and ArcA responses	↑	↓	↓
Pyruvate formate-lyase (PFL) (*focA-pflB*)	↑	↓	↓
PFL repair protein (*grcA*)	↑	↓	↓
Succinate:quinone oxidoreductase (*sdhABCD*)	↓	↑	↑
Aconitate hydratase B (*acnB*)	↓	↑	↑
Cytochrome bo3 ubiquinol oxidase *(cyoA-E)*	n.i.a.	↑	↑
Cytochrome bd-I ubiquinol oxidase (*cydAB*)	↑	↓	↓
Nitrate reductase (*narGHIJ*)	↑	↓	↓
Nitrite reductase (*nirBCD*)	↑	↓	↓
Ni-Fe hydrogenases I, II, III (*hyaABC, hybOABC, hycBCDEFG*)	n.i.a.	↓	↓
**Methionine metabolism**			
Methionine biosynthesis (*metCBHKLN*)	↓	↑	↑
**Putrescine metabolism**			
Putrescine catabolism *(argT, puuAD)*	n.i.a.	↑	↑
Putrescine biosynthesis *(speB)*	↑	↓	___
**Metal ion homeostasis**			
Copper/silver export system *(cusCFBA)*	↑	↓	↓
Iron import *(feoABC)*	↑	___	↓
Iron binding *(ftnB)*	↑	___	↓
Molybdopterin biosynthesis (moaA)	↑	↓	↓
**Oxidative stress and iron-sulfur cluster assembly**			
Catalase/hydroperoxidase HPI *(katG)*	n.i.a.	↑	___
Glutaredoxin 1 (*grxA*)	n.i.a.	↑	___
SUF iron-sulfur assembly system *(sufABCDES)*	n.i.a.	↑	↓*
ISC iron-sulfur assembly system *(iscRSUA-hscBA-fdx)*	n.i.a.	↑	↑

Up and down arrows indicate significant transcript upregulation and downregulation, respectively. N.i.a., no information available. *, most of the SUF operon genes are significantly downregulated.

One of the most striking findings of the transcriptomics analysis of the *tgt* mutant is the altered expression of the FNR and ArcA regulon genes (Fig. 5A and B), indicating that the activity of these two global transcription regulators is possibly repressed in the mutant. FNR and ArcA control the expression of genes that mediate the transition to lower O_2_ levels, FNR being a direct O_2_ sensor while ArcA responds to the redox status of the quinone pool and accumulation of fermentation products.^95^ Predictably, FNR and ArcA activities are repressed in the anaerobic to aerobic transition^93^ while increased in the aerobic to micro-aerobic transition.^94^ FNR and ArcA regulate a great number of genes involved in central metabolism and, as expected, the expression of genes in central metabolism in the *tgt* mutant coincides with what is observed in the anaerobic to aerobic transition (Table 4). Some examples include the repression of the pyruvate formate-lyase (PFL), nitrate and nitrite reductases and Ni-Fe hydrogenases, with concurrent induction of the succinate: quinone oxidoreductase and aconitate hydratase B, as well as the favoring of cytochrome bo3 ubiquinol oxidase (*cyo* genes) over cytochrome bd-I ubiquinol oxidase (*cyd* genes). Changes in expression patterns in methionine biosynthesis and putrescine catabolism genes in the *tgt* mutant also parallels the changes observed in the anaerobic to aerobic transition (Table 4). Partridge *et al.* believed that, along with central metabolism, these biological processes form part of a common core response to changes in O_2_ availability.^94^ A classic OxyR response to oxidative stress, however, is not observed in the *tgt* mutant (e.g. no induction of *grxA* and *katG*). Nonetheless, an induction of the ISC iron-sulfur assembly genes is observed, as seen in the anaerobic to aerobic transition study.^93^ Likewise, the repression of metal ions homeostasis response genes, such as iron import and iron binding, copper export, and molybdopterin biosynthesis genes is observed in the *tgt* mutant, when the expression of these genes was found to be increased in the aerobic to microaerobic transition study.^94^ Based on these comparisons, we concluded that the gene expression changes in the *tgt* mutant resemble the one observed when *E. coli* adapts from low to high O_2_ levels.

### Oxidative stress homeostasis is impacted in the *tgt* mutant

**Fig. 6:**
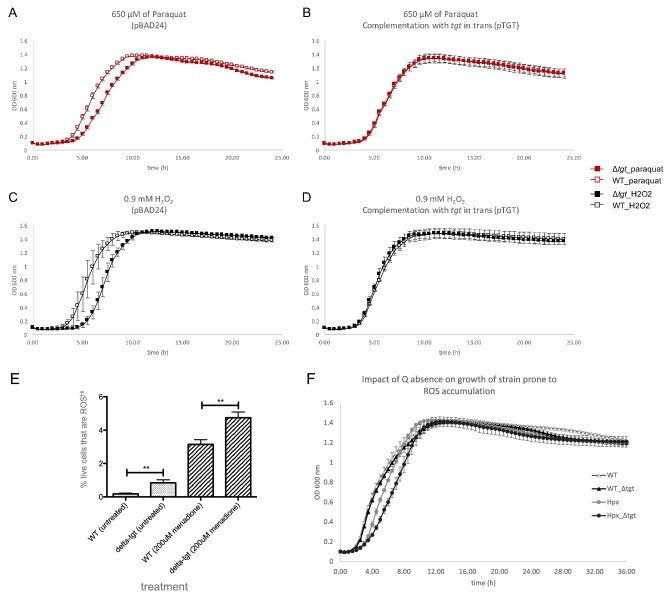
Oxidative stress phenotypes and investigation of ROS levels. (A–D) Growth curves of *E. coli* WT and Δ*tgt* strains monitored in Bioscreen C Analyzer at 37°C with constant shaking. Error bars showing standard deviation for biological triplicates. Both strains were transformed with pBAD24 (*tgt*^–^) (A and C) or pTGT (*tgt*^+^) (B and D) and growth was performed in LB medium supplemented with 100 μg/ml ampicillin and 0.02% arabinose. (A and B) Methyl viologen (paraquat) phenotype at 650 μM. (C and D) Hydrogen peroxide (H_2_O_2_) at 0.9 mM. WT, wild-type. (E) Detection of ROS levels in the *tgt* mutant and wild-type strains. Mean percentage of live cells presenting reactivity to the dye CellROX Green (ROS^HI^). Error bars represent standard error mean (SEM) using four to six biological replicates per experiment. **Significant difference (*P*-value < 0.01). (F) Impact of Q absence on growth of strain prone to ROS accumulation. Growth curves of WT and Hpx^–^ strains, deleted or not for the *tgt* gene. After overnight anaerobic growth, cultures were inoculated in LB media and switched to aerobic environment. Growth was monitored in Bioscreen C Analyzer at 37°C with constant shaking. Error bars show standard deviation for two biological replicates with eight technical replicates each. WT, *E. coli* K12 MG1655; WT_∆tgt, WT deleted for *tgt*; Hpx^–^WT deleted for *katG, katE, ahpC, and ahpF;* Hpx^–^_∆*tgt*, Hpx^–^ deleted for *tgt*.

A consequence of increased aerobic respiration in the cell is the generation of oxidative stress.^96^ Therefore, we tested the sensitivity of the *E. coli tgt* strain to oxidative stressors using different concentrations of hydrogen peroxide (H_2_O_2_) and methyl viologen (also known as paraquat) in LB medium. The *tgt* mutant shows a slight sensitivity to 650 μM paraquat and to 0.9 mM hydrogen peroxide when compared to the WT strain during exponential growth (Fig. 6A and C). And this phenotype was complemented by the overexpression of the *tgt* gene (Fig. 6B and D).

We then looked at levels of ROS in the *tgt* mutant and WT strains, using a technique that couples flow cytometry with the probe CellROX Green to provide an estimation of ROS generation in the cell. Using this probe, high fluorescence quantum yields only upon ROS activation and binding to dsDNA. Untreated cells were compared to cells treated with menadione, a quinone that undergoes catalytic one-electron redox cycling to reduce molecular oxygen (O_2_) to superoxide (O_2_^● –^), thus enhancing ROS detection.^66^ Although subtle, significant and reproducible increase in ROS levels was detected in the *tgt* mutant compared to the WT strain. This result was observed in both untreated and treated conditions and was more prominent after menadione treatment (Fig. 6E, and Fig. S7 in Supplemental file 1). This result corroborates the subtle sensitivity phenotype observed under hydrogen peroxide and methyl viologen stress.

To further investigate the impact of Q absence on ROS accumulation, we deleted the *tgt* gene in the *E. coli* Hpx^–^ strain (*katG katE ahpCF*) and its growth was compared to the original Hpx^–^ strain in LB medium. The Hpx^–^ strain, lacking the three major enzymes responsible for hydrogen peroxide (H_2_O_2_) scavenge, is prone to accumulation of ROS, normally produced in the cell during aerobic growth, and to effects resulting from Fenton reaction, such as DNA damage.^97^ After overnight incubation in anaerobic conditions, the cells were inoculated in LB medium and grown in aerobic conditions in microtiter plates. In this condition, the Hpx^–^∆*tgt* strain grows more poorly than the Hpx^–^ strain, supporting the importance of TGT, thus of Q-modified tRNA, for protection against oxidative stress (Fig. 6F).

In our transcriptomic analysis, the *katG, katE, ahpC, and ahpF* genes were not differentially expressed in the *tgt* mutant in LB. Accordingly, the protein levels of KatG, AhpC, and AhpF were unchanged in the *tgt* mutant compared to the WT. This observation indicates that the absence of Q in tRNA possibly affects another system involved in the protection from or generation of oxidative stress in the cell.

Knowing that a possible consequence of increased hydroxyradicals is the generation of DNA lesions,^96,98^ we tested the sensitivity of the *tgt* mutant to the alkylating agent MMS, and found the *tgt* mutant slightly more sensitive than the WT strain (Fig. S8A in Supplemental file 1). This phenotype was rescued by overexpression of the *tgt* gene in trans (pTGT) (Fig. S8B). To confirm the DNA damage in the cell, we quantified a few DNA oxidation products in the WT and *tgt* mutant strains. Our measurements showed no significant differences in the levels of the analyzed DNA damage products (Fig. S9 in Supplemental file 1) and we concluded that, although there may be elevated ROS in the mutant, this is not causing an increase in the steady-state level of DNA damage products.

### Loss of *tgt* makes *E. coli* more sensitive to aminoglycosides

A few links between Q biosynthesis genes and antibiotics treatment have been already observed. A TnSeq fitness study showed that, in organisms like *Pseudomonas simiae* and *Desulfovibrio vulgaris*, insertions in the Q related genes *tgt, queC, queF*, and *gluQ* are usually detrimental to the growth phenotype under gentamicin stress^67^ [dataset] (https://fit.genomics.lbl.gov/cgi-bin/orthCond.cgi?expGroup=stress&condition1=Gentamicin%20sulfate%20salt). The expression levels of *yhhQ* were increased in *E. coli* exposed to sublethal doses of ampicillin.^99^ And, in *A. baumannii*, levels of the QueC protein were found increased under imipenem exposure, what the authors speculate could help avoid adverse effects on translational fidelity under an antibiotic stress response.^100^

**Fig. 7:**
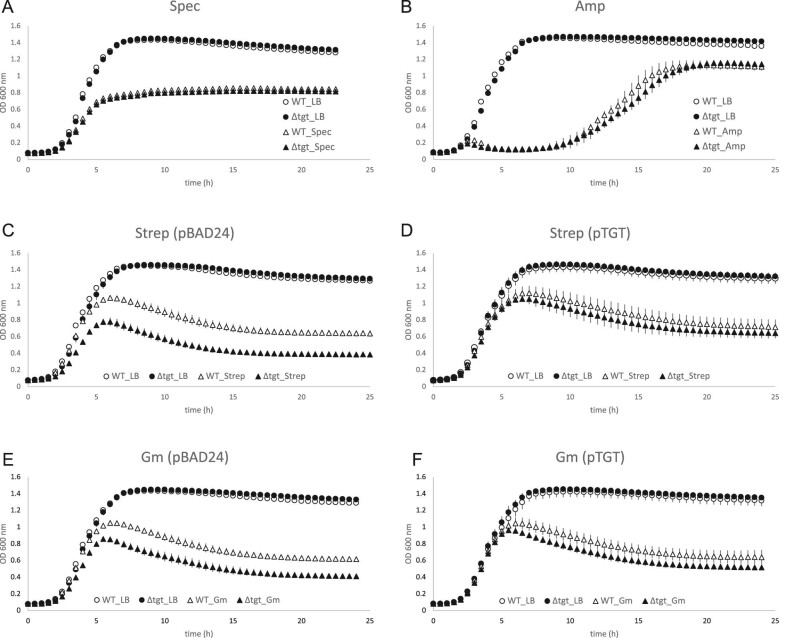
*E*scherichia *coli* Δ*tgt* antibiotics phenotypes. Growth curves of WT and Δ*tgt* strains monitored in Bioscreen C Analyzer at 37°C with constant shaking. Error bars showing standard deviation for five biological replicates. (A–F) In the same graph, curves are shown for growth in LB only and in LB supplemented with a specific antibiotic. (A and B) Strains not transformed with any plasmid. (A) Spectinomycin phenotype at 15 μg/ml Spec. (B) Ampicillin phenotype at 10 μg/ml Amp. (C and E) Strains transformed with pBAD24 (*tgt*^–^); (D and F) strains transformed with pTGT (*tgt*^+^). (C) Streptomycin phenotype at 4 μg/ml Strep. (B) Complementation of streptomycin phenotype by overexpression of *tgt* gene. (C) Gentamycin phenotype at 1 μg/ml Gm. (D) Complementation of gentamycin phenotype by overexpression of *tgt* gene. (C–F) Growth curves were generated in LB medium supplemented with 100 μg/ml ampicillin and 0.02% arabinose for maintenance and expression of plasmids.

Since tRNA modifications directly affect translation, and antibiotics also affect translation in many different ways,^101^ we tested the *tgt* mutant response to antibiotics from different categories. Sensitivity to different concentrations of antibiotics under minimum inhibitory concentration was tested. The sensitivity to spectinomycin and ampicillin was tested in a range of concentrations, but no difference in sensitivity between WT and *tgt* mutant was observed (Fig. 7A and B). The *tgt* mutant shows higher sensitivity to streptomycin (4 μg/ml) and to gentamycin (1 μg/ml) when compared to the WT strain (Fig. 7C and E), and the growth curve pattern for the two antibiotics is the same. This phenotype was partially complemented by the overexpression of the *tgt* gene from the pTGT plasmid (Fig. 7D and F).

Ampicillin is a penicillin beta-lactam antibiotic, whose bactericidal activity results from the inhibition of cell wall synthesis culminating in cell lysis.^102^ Spectinomycin, an aminocyclitol, is a translation inhibitor that does not cause misreading, and that has previously been used as a nonmisreading control.^103,104^ Aminoglycosides, such as streptomycin and gentamycin, cause translational misincorporation and misfolding of proteins.^104^ The fact that the Q absence confers higher sensitivity to streptomycin and gentamycin reinforces the findings by the Farabaugh group that Q absence affects translation accuracy.^23^

Because a connection between iron bioavailability and antibiotic efficacy has been observed,^87^ and because the *tgt* mutant shows a disturbance in its iron homeostasis response (downregulating iron import), we limited iron availability in the growth medium to observe if this would affect the streptomycin phenotype. Using plate assays in MA medium, WT and *tgt* mutant strains were exposed to 2 μg/ml streptomycin under iron-replete (supplemented with 0.001 g/l ammonium iron citrate, CAF) or iron-deplete (no CAF, supplemented with 100 μM of the iron chelator 2,2’-Bipyridyl, BIP) conditions. Under iron-replete condition (Fig. S10A), the increased sensitivity of the *tgt* mutant to streptomycin was confirmed, mainly between 10^–2^ and 10^–5^ dilutions. When iron was depleted with the cell-penetrating iron chelator (that binds unincorporated intracellular iron), the difference in sensitivity to the antibiotic was attenuated in the *tgt* mutant compared to the WT strain (Fig. S10B). This result reveals the importance of iron to the streptomycin sensitivity as well as a difference in intracellular iron levels between the WT and *tgt* mutant strains.

## Discussion

In the current study, we found, among other phenotypes, that the Q-deficient strain (*tgt* mutant) presented increased resistance to the transition metal nickel (Ni) in comparison to an isogenic WT strain. Through a transcriptomics study, the *tgt* mutant resistance to Ni was explained by a repression of the main Ni import system of the cell, the *nikABCDE* operon, even when Ni was not supplemented in the medium. In the *tgt* mutant, the repression of the *nikABCDE* operon seems to be part of a range of bioprocesses whose regulations were disturbed due to an oxidative stress like response. Gene expression regulation in the Q-deficient strain resembles a transition to higher O_2_ levels, with repression of the major regulators FNR and ArcA, and consequent alteration of their regulons causing, for instance, downregulation of nitrate and nitrite reductases and of hydrogenases, and de-repression of genes in the TCA cycle and cytochrome *bo* terminal oxidase complex. An expected consequence of increased aerobic metabolism in the cell is the generation of oxidative stress.^105^ We tested and confirmed that the *E. coli tgt* mutant is more sensitive to the oxidative stressors hydrogen peroxide (H_2_O_2_) and methyl viologen (Paraquat). Detection of ROS in the *tgt* mutant and in the WT strain revealed a slight increase in ROS levels in the mutant, both in the presence and absence of menadione. Finally, we showed that the deletion of the *tgt* gene from a strain prone to ROS accumulation (Hpx^–^) causes it to grow more poorly when anaerobic cultures are aerated.

Despite the evidence of increased ROS levels in the Q-deficient strain, classical responses to oxidative stress through OxyR and SoxRS^106^ were not triggered in the mutant. However, the regulation of other pathways and biological processes signal that a response to increased ROS is happening, including induction of aromatic (Tyr, Trp, Phe) and sulfur amino acids (Met), control of central metabolism (TCA cycle), cofactor synthesis and repair (balance of SUF and ISC systems), folding and refolding of proteins, and a strong control of iron homeostasis through induction of Fur.^107,108^ The regulation triggered by Fur activity is multifactorial, influencing many bioprocesses associated to resistance to oxidative stress such as activation of TCA cycle, induction of the iron superoxide dismutase *sodB* (through RyhB repression) and repression of iron import. Reducing the pool of available iron or limiting its reduction in the cell prevents the occurrence of Fenton and limits DNA damage.^86,96^ In the *tgt* mutant, DNA damage products were not detected and, therefore, we presume that the iron homeostasis control of the cell is sufficient to prevent the Fenton reaction and its severe consequences through hydroxyl radicals.

The Fe-S biosynthesis systems are usually described as ISC, for housekeeping maintenance of the cell, and SUF, induced during oxidative stress or low iron conditions.^109,110^ Despite the observed evidence of increased oxidative stress in the *tgt* mutant, we see an induction of the ISC system and repression of the SUF system, thus not an expected regulation during oxidative stress response. However, a similar regulation was observed in an *E. coli* strain laboratory-evolved to grow on 0.8 mM Paraquat (PQ),^108^ which presented, like the *tgt* mutant, higher expression of *iscRSUA* and *hscBA-fdx-iscX*, and downregulation of the *sufABCDSE* transcription unit. Palsson's group found that ISC is preferable over SUF under ROS stress when the rate of Fe-S cluster inactivation at IscU scaffolds remains below a certain threshold. In that case, a mutation was found at a Cys residue in IscR, what the group suggests could possibly hinder IscR's ability to incorporate 2Fe-2S and explains how the ROS-evolved strain deregulated ISC and SUF expressions.^108^ This could justify why, even with increased ROS levels in the *tgt* mutant, a classic OxyR or SoxRS response is not triggered. If in the *tgt* mutant, like in the strain adapted to increased ROS, the rate of Fe-S cluster inactivation at IscU scaffolds also remains below a certain threshold, this could explain the undetected imbalance in free iron and in Fe-S cluster levels, while still observing the streptonigrin phenotype and a repression of Fur regulated genes. A resultant mutation in IscR, hindering its ability to incorporate 2Fe-2S, could also explain the regulation pointing to an apparent apo-IscR in the *tgt* mutant. Thus, these possible events should be further investigated in the Q-deficient strain.

Previous studies have found connections between tRNA modification defects and oxidative stress both in eukaryotes and prokaryotes. The eukaryotic oxidative stress defects could be originated by different mechanisms as modifications, including Q, have been shown to be determinants for stress-induced ribotoxins.^111^ In some bacteria, such as *P. aeruginosa* PAO1, it has been shown that tRNA modifications like m^7^G46 play a regulatory role in the oxidative stress response by modulating the expression of *katA* and *katB* genes.^112^ In other bacteria, however, such as the Q-deficient *Streptococcus pneumoniae*^26^ and *E. coli* devoid of m^6^A37 in tRNA_1_Val(cmo^5^UAC) (*yfiC* mutant in^113^ or of s^2^U34 (*tusA* mutant in,^114^ no regulatory mechanism has been identified. It is interesting to note that in these last three cases the absence of the tRNA modification gives rise only to modest oxidative sensitivity phenotypes, similar to what we observed in the Q^–^  *E. coli* strain.

**Fig. 8:**
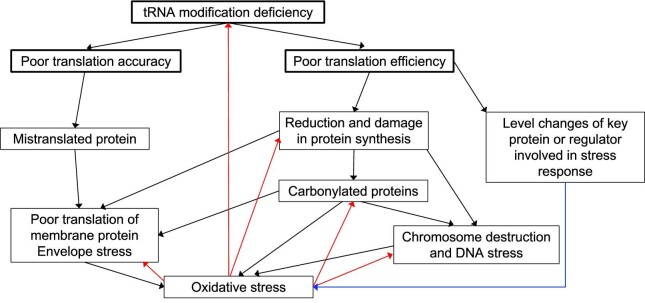
How tRNA modification deficiencies can cause oxidative stress defects. This scheme inspired by Figure 3 of [^115^] shows how changes in tRNA modification levels can affect the oxidative stress phenotypes and responses through different mechanisms. Black arrows show a relationship of cause and effect; red arrows emphasize feedback loops; and blue arrows show possible regulatory mechanisms.

Many indirect mechanisms could be proposed to explain what occurs with the *tgt* mutant strain. However, when considering its main observed phenotypes being associated to metal, aminoglycosides, and oxidative stress, there is abundant fundament in the literature to correlate these events to translation. It has been shown that general translation defects caused by antibiotics or ribotoxins will trigger oxidative stress, mainly through the mistranslation of membrane proteins involved in the electron transfer chain machinery^115–117^ which can, in turn, create a negative feedback loop of protein damage and aggregation.^118^ In Fig. 8, we propose a mechanism for how tRNA modification deficiencies in general can cause oxidative stress defects, with poor translation accuracy and efficiency resulting in mistranslated and damaged proteins, which will originate carbonylated proteins, poor translation of membrane proteins, DNA stress, and ultimately oxidative stress. Regulatory mechanisms resulting from poor translation efficiency might occur with level changes of key proteins or regulators in stress responses. Therefore, considering the plausibility of the Q function in translation, the affected pathways of the *tgt* mutant identified in this study, and in light of the groundwork in the literature, we propose that the phenotypes observed in the *E. coli tgt* strain are a response to mistranslation. Nonetheless, further ribosome profiling experiments will be needed to rule out a more specific regulatory role for Q in this model organism.

## Supplementary Material

mfac065_Supplemental_Files

## Data Availability

The data underlying this article are available in the article and in its online supplementary material. For high throughput data: RNAseq scripts are available at https://github.com/vdclab/RNAseq-analysis RNAseq raw data (fastq files) and processed data have been deposited in NCBI's Gene Expression Omnibus and are accessible through GEO Series accession number GSE181239. Available at https://www.ncbi.nlm.nih.gov/geo/query/acc.cgi?acc=GSE181239 Proteomics data was uploaded to the CHORUS database under ID 3655. Available at https://chorusproject.org/anonymous/download/experiment/2a0b77c87014485a993df01e7744a5a6
