# Supplementary material for: The absence of the queuosine tRNA modification leads to pleiotropic phenotypes revealing perturbations of metal and oxidative stress homeostasis in Escherichia coli K12
Source: Metallomics. 2022 Sep 9;14(9):mfac065. doi: 10.1093/mtomcs/mfac065 (PMC9508795; doi:10.1093/mtomcs/mfac065)
Supplement: mfac065_Supplemental_Files [file mfac065_supplemental_files.zip › Supplemental_file_1_REVISED.pdf]

Supplemental file 1

Supplemental Methods

Bulk tRNA Purification and Q Detection for $tRNA^{Asp}_{GUC}$

E. coli WT and *tgt* mutant strains were grown at 37 °C, 200 rpm, in 100 mL of LB medium under nickel (1.5 and 2.0 mM $NiCl_2$) and streptomycin (4 µg/mL Strep) stress. Approximately 5 mL of cell culture were collected at different time points (2-30 h) and cell pellets were harvested by centrifugation at 1,800 g for 15 min at 15 °C. A similar assay was performed in nickel only (1.5 and 2.0 mM $NiCl_2$) and cells were harvested during and after three 24h-passages in these conditions.

Detection of the presence of Q in tRNA was adapted from a protocol developed by Igloi and Kossel (Igloi & Kössel, 1985) and described in detail by Zallot et al. (Zallot, Yuan, et al., 2017). Briefly, bulk tRNAs were prepared from cell pellets, resuspended in 1 mL of Trizol (Thermo Fisher Scientific) and small RNAs were extracted using Purelink miRNA Isolation kit (Thermo Fisher Scientific). Bulk tRNAs were deacylated by incubation in 100 mM Tris-HCl (pH 9) for 30 min at 37 °C. Then tRNAs were precipitated using ammonium acetate, isopropanol, and linear polyacrylamide as a carrier. The obtained pellet was washed with 70% ethanol, dried in a Vacuum Concentrator System (Labconco) and resuspended in RNase free water. Per lane, 200 ng of tRNAs were resuspended in RNA Loading Dye (NEB) and loaded onto a denaturing 8 M urea, 8% polyacrylamide gel (Thermo Fisher Scientific) containing 0.5% of 3-(Acrylamido)phenylboronic acid (Sigma-Aldrich). The migration was performed at 4 °C in 40 mM Tris, 20 mM acetic acid, and 1 mM EDTA pH 8.3 (1X TAE - Sigma-Aldrich). Migrated tRNAs were transferred onto a Biodyne B Nylon membrane (Thermo Fisher Scientific) at 4 °C for 90 min. The membrane

was baked in an oven for 30 min at 80 °C, and then ultraviolet (UV) irradiated in a UV Crosslinker (Fisher FB-UVXL-1000, Thermo Fisher Scientific) at a preset UV energy dosage of 120 mJ/cm^2. $tRNA^{Asp}{}_{GUC}$ was detected with the North2South Chemiluminescent Hybridization and Detection Kit (Thermo Fisher Scientific,Waltham, MA, USA). The initial membrane blocking was realized with DIG Easy Hyb (Roche, Basel, Switzerland). Hybridization was done at 60°C, while using the specific biotinylated primer for $tRNA^{Asp}{}_{GUC}$ (Zallot, Yuan, et al., 2017) (50-biotin-CCCTCGGTGACAGGCAGG-30) at 0.3 µM final. The blot was exposed to X-Ray film (Thermo Scientific, CL-X Posure Film) for 5-30 min and the films were developed using a film processor (Konica QX-60A, Tokyo, Japan).

Determination of Free Iron Concentration

Free iron concentration was determined following the protocol by James Imlay (Keyer & Imlay, 1996). Biological triplicates of overnight cultures of WT and *tgt* mutant strains were subcultured in LB medium, grown at 37 °C, 200 rpm to reach an OD_{600} of 0.90-0.97. Cells were collected by centrifugation of 250 mL at 10,000 g, 10 °C, 20 min (Sorvall GS-3 SLA-3000) and then resuspended in 10 mL of pre-warmed LB medium containing 10 mM diethylenetriaminepentaacetic acid (DTPA) and 20 mM desferrioxamine (DFO). The suspension was incubated at 37°C, shaking at 200 rpm for 15 minutes. DTPA blocks further iron import during this period while DFO penetrates cells, binds ferrous iron and, in the presence of oxygen, oxidizes the iron to the ferric form. Usually, ferric iron, but not ferrous iron, is visible by perpendicular mode X-band EPR. Therefore, this method quantifies any iron that DFO can bind in the cell. After

centrifugation (3,000 g, 4°C, 15 min), the pellet was washed twice with ice-cold 20 mM Tris-HCl (pH 7.4) and finally resuspended in ice-cold 20 mM Tris-HCl (pH 7.4) containing 10% glycerol. 400 µL of sample was added into a quartz EPR tube (4×5 mm ID×OD) and immediately frozen on dry ice. A standard curve was prepared with iron(III) chloride hexahydrate ($FeCl_3 \cdot 6H_2O$) in 20 mM Tris-Cl pH 7.4, 10% glycerol, 1 mM desferrioxamine. OD_{420} of each dilution was measured and the extinction coefficient (2865 M^{-1} cm^{-1} for Fe/DFO) used to calculate the actual iron concentration.

EPR spectra were recorded on a Bruker Elexsys E500 instrument using an Oxford ESR900 cryostat. Unless otherwise noted, the temperature was set to 35 K. A Bruker ER4116-DM dual mode resonator was used in perpendicular mode. The microwave frequency was 9.41 GHz at 2 mW of power. The modulation frequency was 100 kHz and the modulation amplitude 10 G. To reduce spectral noise, eight scans were averaged for each spectrum. The quantitative assessment of the Fe(III) content of the biological samples was performed by comparing the EPR intensity with the standard curve. Calculations of intracellular iron concentration were made by normalizing iron measurements to intracellular volume, using the conversion that 1 mL of 1 OD bacteria collectively contains 0.5 µL of cytosol.

Beta-galactosidase assays

Beta-galactosidase assay method was adapted from (Miller, 1972; Zhang & Bremer, 1995). Biological triplicates of *E. coli* strains were grown overnight in LB medium at 37 °C, shaking at 200 rpm. Saturated cultures were diluted to OD_{600} of 0.05 in LB medium and grown to reach early exponential phase (about 2 h at 37 °C, 200 rpm). Per

sample, 0.5 mL culture were added by 0.5 mL of Z Buffer (0.06 M Na_2HPO_4, 0.04 M NaH_2PO_4, 0.01 M KCl, 0.001 M $MgSO_4$, pH 7.0, added by β-mercaptoethanol 0.27 mL for 100 mL), 100 µL chloroform, 50 µL 0.01% SDS, vortexed for 30 s, and incubated at 30 °C for 20 min. After permeabilization, 0.2 mL of *o*-nitrophenyl-β-D-galactopyranoside (ONPG) was added per sample and reaction happened at 30 °C until the development of yellow color was observed. Reaction was stopped with 0.5 mL of 1 M Na_2CO_3 and samples were centrifuged at 25,000 g for 1 min at ambient temperature. The absorbance of the upper phase (200 µL) was read at 420 nm and 550 nm. Beta-galactosidase activity, in Miller units, was calculated according to the formula:
1000 X [A_{420}-(1.75*A_{550})]/(vol. of culture in mL X minutes of reaction X A_{600}). All strains used for beta-galactosidase assays are described in Supplemental File 1, Table S1.

Quantification of DNA damage products

Using an adaptation of the analytical method described by Taghizadeh et al. (Taghizadeh et al., 2008), the genomic DNA of *E. coli* WT and *tgt* mutant strains, in biological triplicates, was extracted by the phenol-chlorophorm method (Thiaville et al., 2016) and used to quantify the following DNA oxidation products: N^2-carboxymethyl-2'-deoxyguanosine (CMdG), 8-oxo-2'-deoxyguanosine (8oxodG), 1,N^6-etheno-2'-deoxyadenosine (1,N^6-εdA), and 1,N^2-etheno-2'-deoxyguanosine (1,N^2-εdG). 50 µg genomic DNA was reconstituted in 10 mM Tris-HCl buffer pH 7.9, 1 mM $MgCl_2$, 10 µg/ml coformycin, 50 µg/ml tetrahydrouridine, 1 mM desferrioxamine, and 1 mM butylated hydroxytoluene and digested with 10 U benzonase, 5 U DNase I, 17 U Alkaline phosphatase, 0.1 U phosphodiesterase I. Then, the mixture of isotope labeled standards

containing 0.4 pmol [$^{15}N_5$]-N^2-CMdG, 0.5 pmol [$^{15}N_5$]-8-oxodG, 30 fmol [$^{13}C_{10}$]-1,N^2-εdG, and 15 fmol [$^{15}N_3$]-1,N^6-εdA was spiked immediately after addition of the enzymes. The digestion was allowed to occur for 12 h at 37 °C. The reaction was passed through 10 kDa exclusion filter to remove proteins. The collected filtrates were evaporated and reconstituted in water and a portion analyzed by LC-ESI-MS/MS using an Agilent 1290 series HPLC system interfaced with an Agilent 6490 triple quadrupole mass spectrometer. Samples were resolved on a Kinetex EVO C18 column (2.6 μm, 2.1 × 150 mm, Phenomenex), using 0.1% formic acid in water (solvent A) and acetonitrile (solvent B) delivered at 0% B for 2 min; increased to 16% B for 15 min; increase to 80% B in 1 min and hold at 80% B for 5 min; decrease to 0% B in 1 min and re-equilibrated for 5 min. The flow rate used is 0.400 mL/min. The effluent from the first 1 min from the LC system was diverted to waste to minimize the contamination of the ESI source. The MS was operated in the positive ion mode. Operating parameters were as follows: ESI capillary voltage, 4000 V; gas temperature, 200 °C; drying gas flow, 12 L/min; nebulizer pressure, 30 psi. Samples were analyzed in multiple reaction monitoring (MRM) mode, with the following transitions: *m/z* 326→210 for N^2-CMdG and *m/z* 331→215 for [$^{15}N_5$]-N^2-CMdG; *m/z* 284→168 for 8-oxodG and *m/z* 289→173 for [$^{15}N_5$]-8-oxodG; *m/z* 276→160 for 1,N^6-εdA and *m/z* 279→163 for [$^{15}N_3$]-1,N^6-εdA; *m/z* 292→176 for 1,N^2-εdG and *m/z* 302→181 for [$^{13}C_{10}$]-1,N^2-εdG. Calibration curves for the labeled and unlabeled forms were constructed by plotting the MRM signal ratios between the labeled and unlabeled forms against their corresponding concentration ratios. Quantitation in each sample was

achieved using the MRM signal ratio between analyte of interest and its isotope-labeled internal standard and the response curve.

Supplemental Results

The Ni^{2+} resistance phenotype is not caused by accumulation of Q precursors

To investigate if the nickel resistance phenotype of the Δ*tgt* mutant was caused by the accumulation of Q precursors ($preQ_0$ and $preQ_1$) and not by the absence of Q in tRNA, we analyzed the resistance to nickel stress of strains deleted for the genes *queD* and *yhhQ*, involved in the synthesis and salvage of Q precursors, respectively (Hutinet et al., 2017; Zallot, Yuan, et al., 2017). These mutants grew similarly to the WT strain in LB medium (data not shown). When exposed to 2.5 mM $NiCl_2$, the Δ*queD* Δ*tgt* and Δ*yhhQ* Δ*queD* Δ*tgt* mutants were as resistant as the Δ*tgt* mutant (Figure S1 A and B), confirming that the nickel phenotype is not caused by the accumulation of Q precursors in the cell, but by the absence of Q in tRNA. A Δ*queD* mutant was found to be as sensitive as the WT strain (Figure S1 C). However, these experiments were performed in LB medium, that might contain the Q precursor, which can be salvaged in an *yhhQ*$^{+}$ strain (Yuan et al., 2019).

Fur and iron levels are not altered in the tgt mutant

Because Fur is activated by Fe^{2+} (Seo et al., 2014), the observed expression profiles could be a result of overexpression of Fur and/or increased intracellular iron levels. No differences in the levels of the *fur* gene expression were observed in the *tgt*

mutant (Supplemental file S2) and no differences in protein levels were observed by Western blot analysis using anti-Fur antibodies (Figure S5 A). We then tested if the *ryhB* promoter (P_{ryhB}) was repressed in the *tgt* mutant by using a beta-galactosidase assay, measuring the activity of the enzyme expressed from a fusion of the *ryhB* promoter (P_{ryhB}) inserted upstream of the *lacZ* gene at the original lac site. Despite the strong evidence of *ryhB* repression seen in the RNA-seq transcriptomics data, no difference in promoter activity between WT and *tgt* mutant was observed in the beta-galactosidase assay (Figure S5 B). To investigate if intracellular iron levels were increased in the *tgt* mutant, we tested the strains sensitivity to streptonigrin (SNG), and also measured intracellular iron levels using Electron Paramagnetic Resonance (EPR). Streptonigrin is an iron-activated antibiotic; therefore, high iron-levels will result in stronger sensitivity to this antibiotic (Ezraty & Barras, 2016). Results show that the *tgt* mutant is slightly more sensitive than WT to 0.75 µg/mL SNG (Figure S5 C). Although subtle, this phenotype is consistent through repeated experiments and it was complemented by the overexpression of the *tgt* gene in trans. However, the difference in iron levels that might be causing this phenotype could not be detected by EPR. The EPR experiment measured the combined levels of intracellular Fe^{2+} and Fe^{3+} in the WT and *tgt* mutant strains grown in LB medium, in triplicates. This experiment was repeated twice with similar results, with no significant difference in free iron levels between the WT and the *tgt* mutant (Figure S5 D).

No Fe-S cluster imbalance was observed in the *tgt* mutant

In order to test if there was an imbalance in Fe-S cluster levels in the *tgt* mutant, we measured the activities of promoters regulated by Fe-S proteins. *E. coli* strains, with

and without the *tgt* gene, carrying the *lacZ* reporter gene fused to three different promoters, were tested by beta-galactosidase activity assay. The *iscR* promoter (P_{iscR}) is repressed by Fe-S bound IscR (Vinella et al., 2013); the *hmpA* promoter (P_{hmpA}) is repressed by the Fe-S bound form of NsrR (Vinella et al., 2013); and the *ydfZ* promoter (P_{ydfZ}) is activated by the Fe-S bound form of FNR (Roche et al., 2015). The strain containing the P*ydfZ*-*lacZ* fusion has a mutated version of *fnr* (Δ2-15) whose FNR activity is more stable to O_2 than the WT protein. We found no expressive difference between WT and the *tgt* mutant regarding any of the promoter activities (Figure S6). There was a small difference for the P_{hmpA}, where the WT activity was 4.8 Miller units higher ($P<0.05$) than in the *tgt* mutant. However, because this was a small difference, and not confirmed by the other strains, we concluded that there was no Fe-S cluster imbalance in the *tgt* mutant that could be detected by this method.

Investigation of DNA damage in the *tgt* mutant

Hydroxyradicals can oxidize the ribose or the base moieties of a DNA molecule and cause DNA lesions (Mendoza-Chamizo et al., 2018; van Houten et al., 2018). Because of the observed oxidative stress phenotypes and detected increase in ROS, we tested the sensitivity of the *tgt* mutant to methyl methane sulfonate (MMS), a classical S_N2-type alkylating agent (Baek et al., 2009; Sikora et al., 2015). Between 0.045% and 0.065% MMS concentrations, the *tgt* mutant was found slightly more sensitive than the WT strain (Figure S8). This phenotype was rescued by overexpression of the *tgt* gene in trans (pTGT).

Transcriptomics analysis of the *tgt* mutant grown in LB medium, shows that a few genes related to DNA repair are up-regulated (such as *mfd, xthA* and *mutM*) while others are down-regulated (like *ogt* and *rusA*) (Supplemental file 2). Interestingly, the protein levels of two of the main enzymes responsible for repair of DNA lesions, PolB and AlkB (Berardini et al., 1999; Mielecki & Grzesiuk, 2014), are increased in the total extract of the *tgt* mutant (Table 3 in main text). *MutM*, is one of the genes with discordant RNAseq and proteomics expression results, with up-regulated gene expression ($\log_2$FC 2.36) and reduced protein levels ($\log_2$FC -1.07) (Table 3 and Table 4). MutM works in the base excision repair (BER) pathway to repair the 8-oxoG type of free radical induced DNA lesions, one of the most common lesions produced by HO• (Mendoza-Chamizo et al., 2018; van Houten et al., 2018).

To test the hypothesis that increased oxidative stress in the *tgt* mutant leads to DNA damage, we used isotope labeled standards measured by LC-ESI-MS/MS to quantify the following DNA oxidation products in the WT and *tgt* mutant strains: N^2-carboxymethyl-2'-deoxyguanosine (CMdG), 8-oxo-2'-deoxyguanosine (8-oxodG), 1,N^6-etheno-2'-deoxyadenosine (1,N^6-εdA), and 1,N^2-etheno-2'-deoxyguanosine (1,N^2-εdG). Our measurements showed no significant differences in the levels of the analyzed DNA damage products between the *tgt* mutant and WT cells (Figure S9). We conclude that, although there may be elevated ROS in the mutant, this is not causing an increase in the steady-state level of DNA damage products.

Supplemental Figures

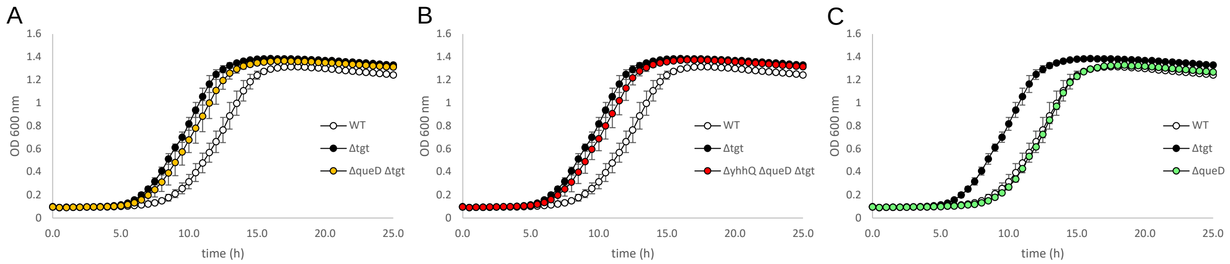

Figure S1. Nickel phenotype of *E. coli* mutants for genes involved in the synthesis and salvage of Q precursors. Growth of *E. coli* WT, Δ*tgt* (no Q in tRNA but accumulation of preQ$_0$ and preQ$_1$ precursors), Δ*queD* Δ*tgt* (no Q in tRNA and no synthesis, but salvage of preQ$_0$ and preQ$_1$ precursors), Δ*yhhQ* Δ*queD* Δ*tgt* (no Q in tRNA, neither synthesis nor salvage of preQ$_0$ and preQ$_1$ precursors) and Δ*queD* (no synthesis, but salvage of preQ$_0$ and preQ$_1$ precursors) strains monitored in Bioscreen C Analyzer at 37°C with constant shaking. Error bars showing standard deviation for biological triplicates. Growth was performed in LB medium supplemented with 2.5 mM $NiCl_2$.

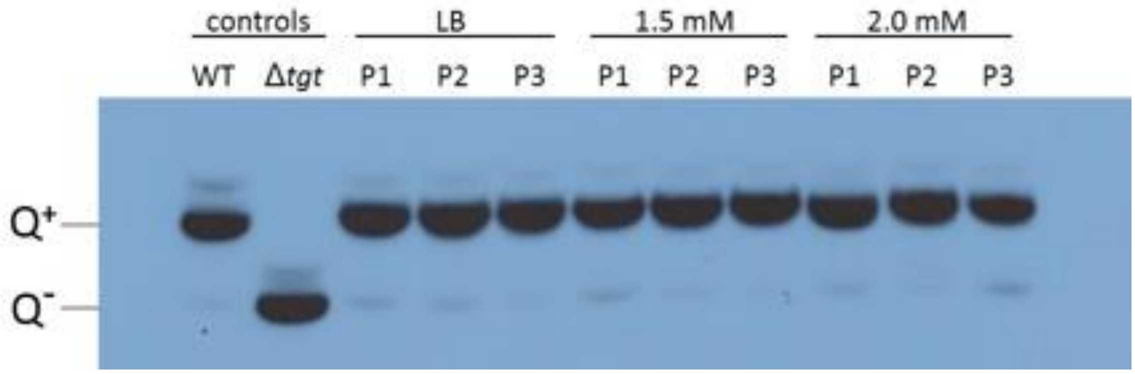

Figure S2. Detection of Q modification levels in $tRNA^{Asp}_{GUC}$ under nickel exposure. *E. coli* MG1655 bulk tRNAs were collected after 24-hour passages and separated in an 8 M urea, 8% polyacrylamide gel containing 0.5% 3-(acrylamido)phenylboronic acid and then transferred to a nylon membrane. The transferred tRNAs were probed with a

biotinylated primer, and visualized by chemiluminescence. tRNAs modified with Q (**Q+**) migrate slower than unmodified tRNAs (**Q-**), as illustrated with tRNA from wild-type (WT) and Δ*tgt* grown in Luria-Bertani (LB - positive and negative control, respectively). Samples of WT strain were collected after passages in LB only and LB supplemented with 1.5 or 2.0 mM $NiCl_2$. P1, passage day 1; P2, passage day 2; P3, passage day 3.

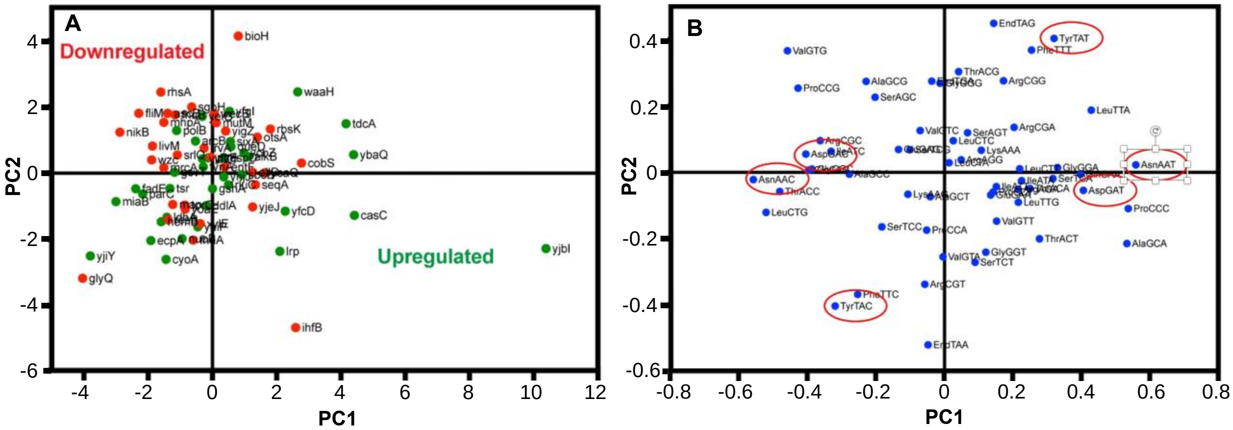

Figure S3. Principal components analysis (PCA) of Q-dependent protein changes and codon usage in the *E. coli tgt* mutant compared to wild-type. The graphs show the (**A**) scores and (**B**) loading plots for PCA analysis of codon usage values for the top 37 up- and 33 down-regulated proteins in the *E. coli tgt* mutant compared to wild-type. Red circles denote Q-dependent codons; His codons are not clearly resolved in the plot and not indicated with red circles.

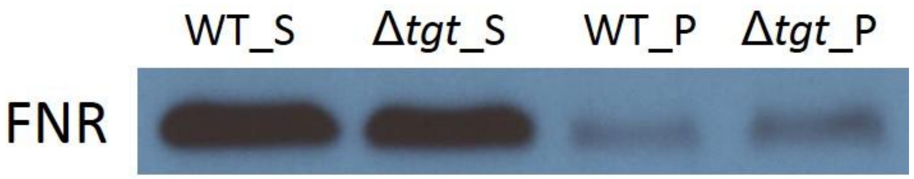

Figure S4. Expression of endogenous FNR protein in WT and *tgt E. coli* strains. Cell cultures were collected at early exponential phase (OD_{600} ~0.6) and protein extracts prepared using a French Press followed by ultracentrifugation. Western blot was performed in PVDF membrane with detection of FNR proteins by rabbit ant-FNR

antibodies. WT, wild-type; Δ*tgt*, *tgt* mutant; S, soluble fraction; P, aggregate ("pellet") fraction; FNR, fumarate and nitrate reduction regulatory protein.

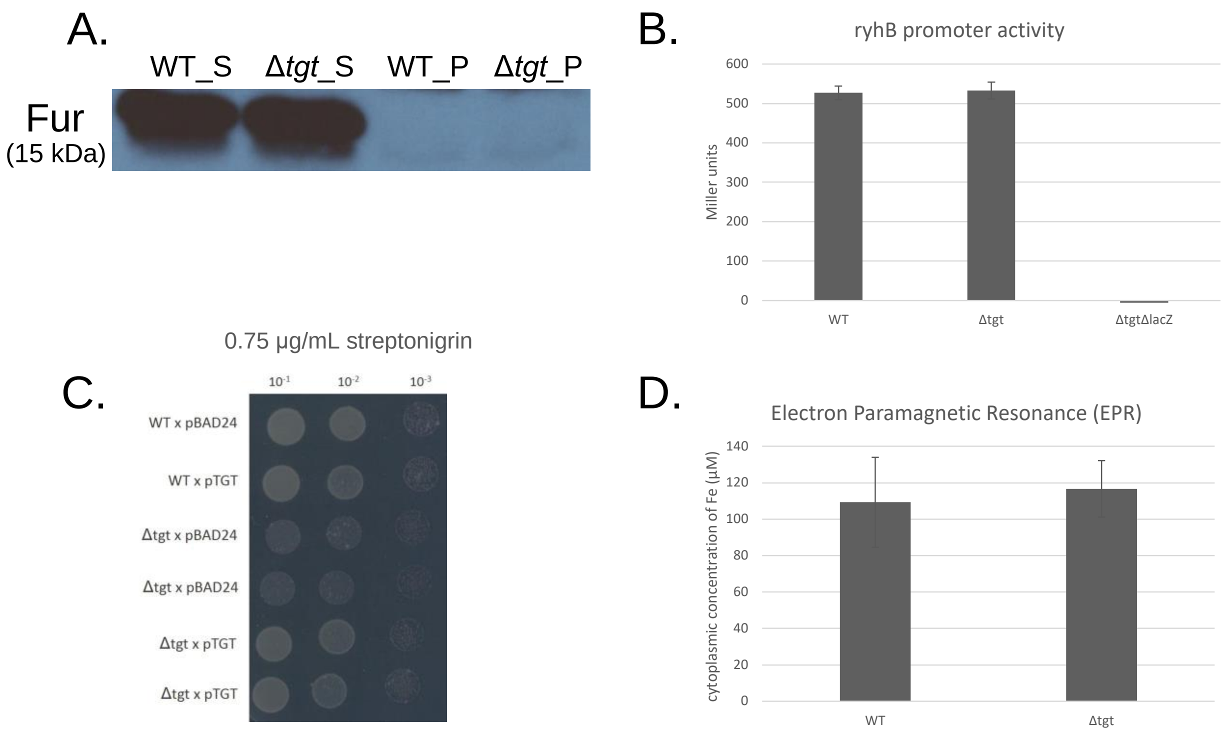

Figure S5. Investigation of Fur regulation and iron levels in the *tgt* mutant. A, Western blot of *E. coli* WT and *tgt* mutant soluble (S) and insoluble (P) protein extracts using anti-Fur antibodies. B, β–galactosidase activity assay of *lacZ* gene fused to the *ryhB* promoter (inserted at the original lac site) expressed in WT and *tgt* strains; Δ*tgt*Δ*lacZ* strain does not express the fusion and was included as a negative control. Error bars representing five biological replicates. C, sensitivity to the iron-activated antibiotic streptonigrin. WT and *tgt* strains transformed with pBAD24 or pTGT (*tgt* cloned into pBAD24). Drops from serially diluted cultures in MA medium supplemented with 0.75 µg/mL streptonigrin, 100 µg/mL ampicillin, 0.02% arabinose. D, Electron Paramagnetic Resonance (EPR) for detection of combined levels of intracellular Fe^{2+} and Fe^{3+} in *tgt* and WT strains grown in LB medium. Error bars representing biological triplicates.

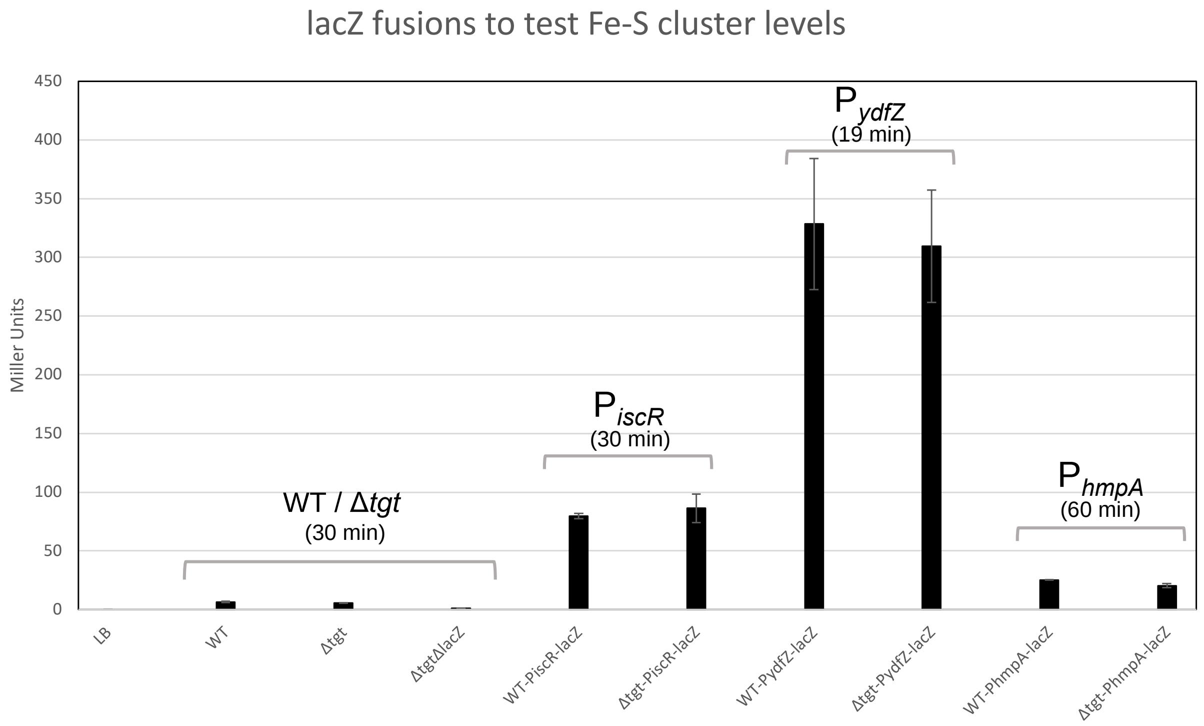

Figure S6. Investigation of Fe-S cluster imbalance in the Q$^-$ strain. Beta-galactosidase activity assay of *lacZ* gene fused to the promoters P_{iscR} (repressed by holo-IscR), P_{ydfZ} (activated by the Fe-S bound form of FNR), and P_{hmpA} (repressed by the Fe-S bound form of NsrR) in WT and Δ*tgt* strains. Strains not containing the *lacZ* fusions were used as controls; WT and Δ*tgt* contain the regular *lacZ* gene while Δ*tgt*Δ*lacZ* does not. Error bars representing three biological replicates.

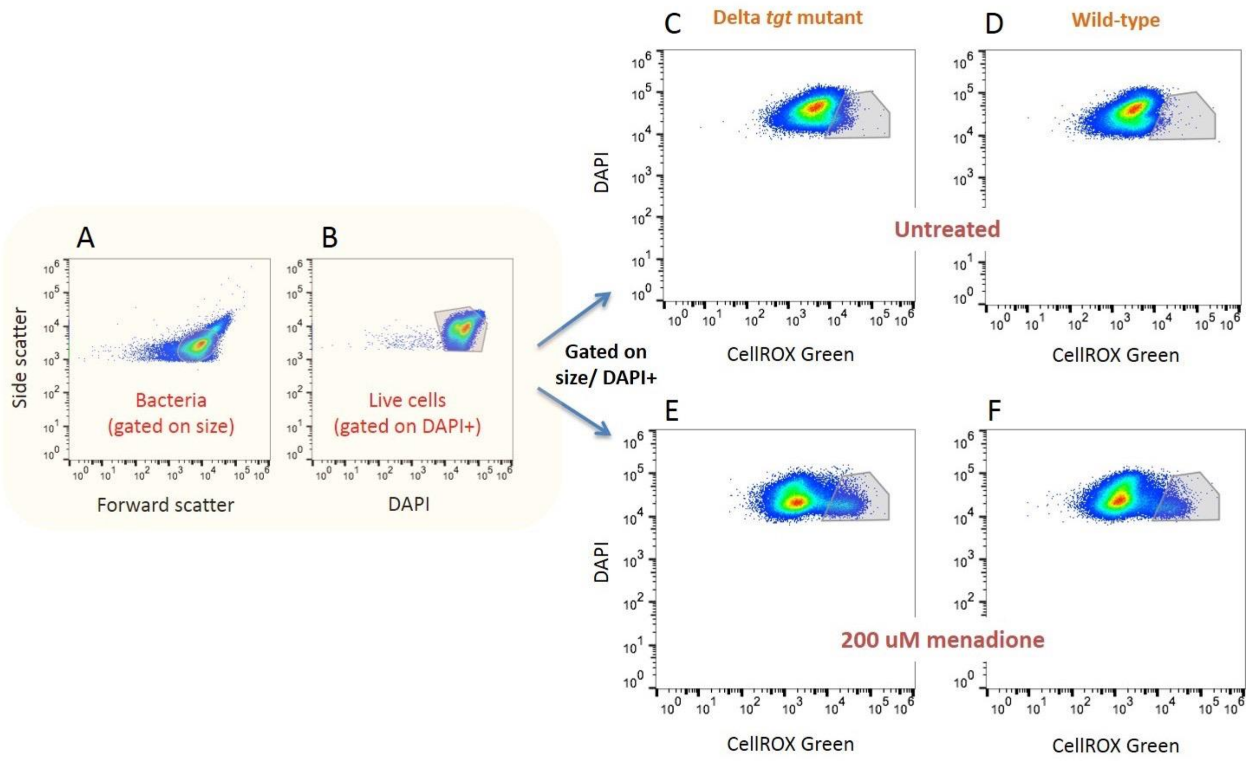

Figure S7. Detection of ROS levels by flow cytometry coupled with fluorescent dye. Representative contour plots of the gating scheme of *E. coli* K-12 MG1655. **A** and **B**, selection based on size and DAPI staining, respectively. **C, D, E, F**, cells selected for dye reactivity (CellROX Green). **C** and **D**, *tgt* mutant and wild-type untreated cells. **E** and **F**, *tgt* mutant and wild-type cells treated with menadione.

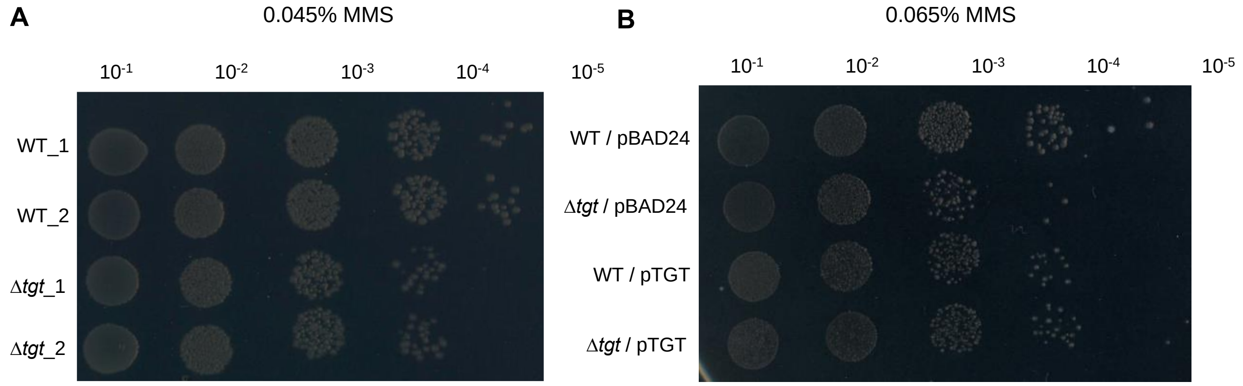

Figure S8. Sensitivity to methyl methane sulfonate (MMS). **A**, WT and *tgt* strains, in biological duplicates, grown in LB medium supplemented with 0.045% MMS for 20 h at 37ºC. **B**, WT and *tgt* strains transformed with pBAD24 or pTGT (*tgt* cloned into pBAD24) grown in LB medium supplemented with 0.065% MMS, 100 µg/mL ampicillin and 0.02% arabinose for 20 h at 37ºC. For the experiments, overnight cultures grown in LB medium (A) or LB supplemented with 100 µg/mL ampicillin (B) were diluted and grown to mid-exponential phase, then diluted to OD(A_{600nm}) 1.0, and 7 µL of 10-fold serial dilutions were spotted on the respective plates.

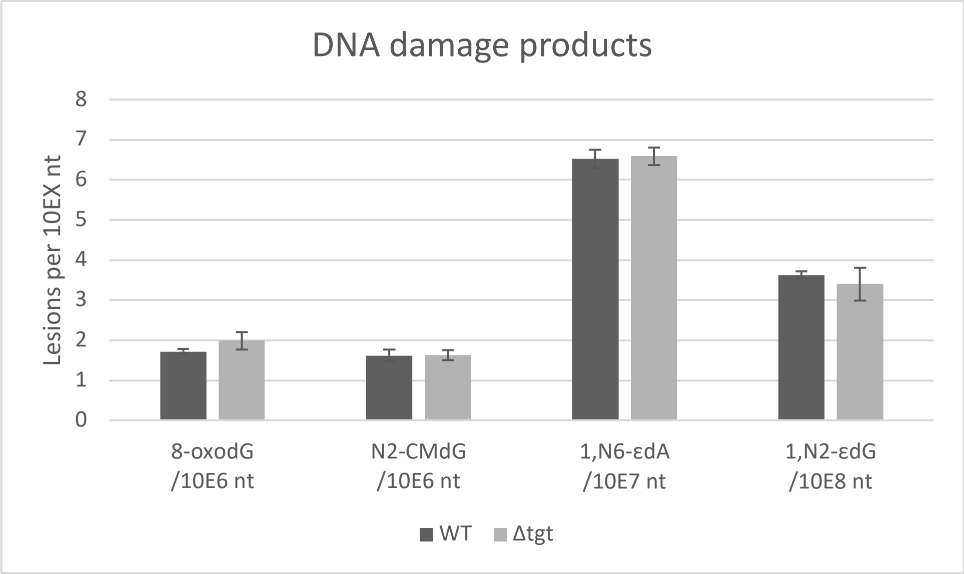

Figure S9. Quantification of DNA oxidation products. N^2-Carboxymethyl-2'-deoxyguanosine (CMdG), 8-oxo-2'-deoxyguanosine (8-oxodG), 1,N^6-etheno-2'-deoxyadenosine (1,N^6-εdA), and 1,N^2-etheno-2'-deoxyguanosine (1,N^2-εdG) were quantified in genomic DNA from WT and *tgt* mutant strains using isotope labeled standards measured by LC-ESI-MS/MS. DNA was isolated from cells grown in LB medium to mid exponential phase. Data are depicted as mean ± SD for 3 experimental replicates.

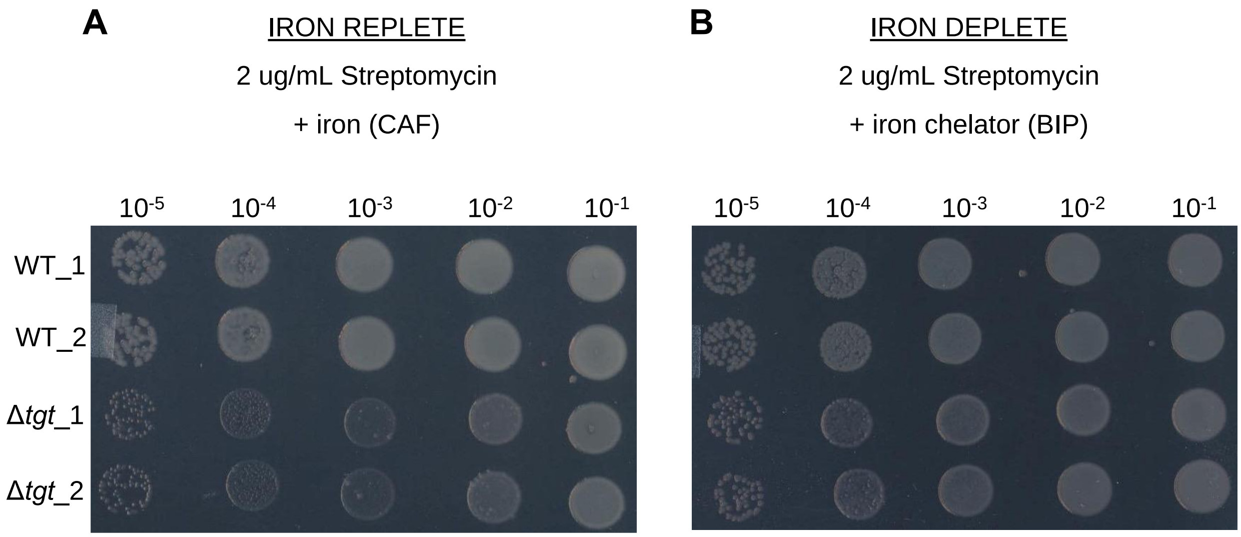

Figure S10. Attenuation of the *E. coli* Δ*tgt* streptomycin phenotype by addition of iron chelator. A and **B**, WT and *tgt* strains, in duplicates, grown at 37ºC for 48 h in MA medium with double the regular $MgSO_4$ concentration (3.65 mM) and supplemented with 0.2% glycerol. **A**, iron replete condition: supplemented with 0.001 g/L of ammonium iron citrate (CAF). **B**, iron deplete condition: no added CAF and supplemented with 100 µM of the iron chelator 2,2'-Bipyridyl (BIP). For the experiments, overnight cultures grown in MA medium supplemented with 0.001 g/L CAF were diluted to OD(A_{600nm}) 0.2 and grown for five hours (reaching optical density around 1.0). Cultures were normalized to the same OD and then 7 µL of 10-fold serial dilutions were spotted on the respective plates.

Supplemental Tables

Table S1. Strains and plasmids used in this study

Name	Code	Genotype or characteristics	Reference
**Strains**			
MG1655	MG1655	F- λ- ilvG- rfb-50 rph-1	(Blattner et al., 1997)
BW25113	BW25113	[Δ(araD-araB)567 Δ(rhaD-rhaB)568 ΔlacZ4787 (::rrnB-3) hsdR514 rph-1]	(Grenier et al., 2014)
Δ*tgt*	VDC4548	As BW25113 plus Δ*tgt::kan*	this study
Δ*tgt*	VDC4561	As MG1655 plus Δ*tgt::kan*	this study
Δ*tgt*	LPO0126-LPO0128	As MG1655 plus Δ*tgt::kan*	this study
Δ*tgt* Δ*lacZ*	VDC4560	As Δ*tgt* plus Δ*lacZ*	this study
Δ*queD*	VDC2043	As MG1655 plus Δ*queD::kan*	(Phillips et al., 2012)
Δ*queD* Δ*tgt*	LPO0120-LPO0125	As MG1655 plus Δ*queD* Δ*tgt::kan*	this study
Δ*yhhQ* Δ*queD* Δ*tgt*	VDC4584	As MG1655 plus Δ*yhhQ* Δ*queD* Δ*tgt::kan*	this study
P*ryhB::lacZ*	PM2230	PryhB-lacZ, mal::lacIq, ΔaraBAD	A gift from Pierre Mandin
P*ryhB::lacZ* Δ*tgt*	LPO0110-LPO0112	As PM2230 plus Δ*tgt::kan*	this study
P*iscR::lacZ*	DV901	Δ*lacZ* P*iscR*::*lacZ*	(Vinella et al., 2013)
P*iscR::lacZ* Δ*tgt*	LPO0101-LPO0103	As DV901 plus Δ*tgt::kan*	this study
P*hmpA::lacZ*	DV1301	ΔlacZ PhmpA::lacZ	(Vinella et al., 2013)
P*hmpA::lacZ* Δ*tgt*	LPO0107-LPO0109	As DV1301 plus Δ*tgt::kan*	this study
P*ydfZ::lacZ*	BR616	*lacZ*Δ145 λ - P*ydfz-lacZ* (SpecR) bla::(Δ2-15)-*fnr*	(Roche et al., 2015)
P*ydfZ::lacZ* Δ*tgt*	LPO0095-LPO0097	As BR616 plus Δ*tgt::kan*	this study
Hpx^-	LC106	As MG1655 plus Δ(ahpC-ahpF′) kan::′ahpF Δ(katG17::Tn10)1 Δ(katE12::Tn10)1	(Seaver & Imlay, 2004)
Δ*tgt*	LPO0140-LPO0147	As MG1655 plus Δ*tgt::cat*	this study
Hpx^- Δ*tgt*	LPO0148-LPO0155	As Hpx^- plus Δ*tgt::cat*	this study
GC10	GC10	F- mcrA Δ(mrr-hsdRMS-mcrBC) φ80dlacZΔM15 ΔlacX74 endA1 recA1 Δ(ara, leu)7697 araD139 galU galK nupG rpsL λ- T1R	Gene Choice
**Plasmids**			
pBAD24	pBAD24	AmpR, ColE1, arabinose inducible promoter	(Guzman et al., 1995)
pTGT	pCH111	*E. coli tgt* gene inserted in pBAD24	(Hutinet et al., 2019)

Table S2. Oligonucleotide primers used in this study

Primer code	Primer name	Orientation	5' - 3' sequence
**Deletion of *tgt* gene**			
**CH316**	tgt_deletion_F	Fwd	ATGAAATTTGAACTGGACACCACCGACGGTCGCGCAC<br>GCCGTGGCCGCCTATTCCGGGGATCCGTCGACC
**CH317**	tgt_deletion_R	Rev	ACGGTCAAGATGATGCAAGTAAGCGCGTGAATAATTG<br>CGACAGGTGTAGCTGTAGGCTGGAGCTGCTTCG
**To check absence of *tgt* gene**			
**JMB93**	tgt_ext_F	Fwd	ACGAAGACGCTGACCGCGAACTTTATCAAACCG
**JMB94**	tgt_ext_R	Rev	GGGCAAGGTTAAGCGCCACGACGTATTTGTCACCC
**JMB95**	tgt_int_F	Fwd	GAAATCATGAAACTGCACGGCGATCTGCAC
**JMB96**	tgt_int_R	Rev	GCAAGTAAGCGCGTGAATAATTGCGACAGG
**To amplify *fnr* from WT and *tgt* mutant strains**			
**LPO21**	fnr_F	Fwd	AATTACGGCTTGAGCAGACC
**LPO22**	fnr_R	Rev	AAGGATAGTGAGTTATGCGGAAAAA
**To construct P*ryhB::lacZ* PM2230 (by P. Mandin)**			
**PryhB-300-F**	PryhB-300-F	Fwd	CGAAGCGGCATGCATTTACGTTGACACCATCGAATGG<br>CGCCCATCAATACAGGCGACGAG
**PryhB-lacZ-R**	PryhB-lacZ-R	Rev	TAACGCCAGGGTTTTCCCAGTCACGACGTTGTAAAAC<br>GACCATAGCTGTTTCCTGTGTGACGAGACAATAATAAT<br>CATTC
**To check *lacZ* fusions**			
**LPO24**	piscR_F	Fwd	GCATCCGACAACAGGTACAA
**LPO25**	phmpA_F	Fwd	TCATTGTGCGATAACAGGTCTT
**LPO26**	pdfZ_F	Fwd	TTCATTTCCTCTCATCCCATCC
**LPO30**	pryhB_F	Fwd	GATCCAACAATTTCACACAGGAAAC
**LPO27**	lacZ_int_R	Rev	CAAAGACCAGACCGTTCATACA
**To construct Hpx$^-$ Δ*tgt***			
**YY190**	Eco_tgt_del_F	Fwd	ATGAAATTTGAACTGGACACCACCGACGGTCGCGCAC<br>GCCGTGGCCGCCTGGTGTAGGCTGGAGCTGCTTCG
**YY191**	Eco_tgt_del_R	Rev	TTAATCAACGTTCAAAGGTGGTACTTCTCGCCCC<br>CTGACGCTGGTAAAAATCGTCCATATGAATATCC<br>TCCTTAG

Table S3. Fitness heatmap for genes in Q pathway in different conditions

Data obtained from Fitness Browser website http://fit.genomics.lbl.gov/cgi-bin/myFrontPage.cgi (Price et al., 2018) showing top 30 experiments, sorted by average fitness. Fitness values (as described on Fitness Browser): Gene fitness is the weighted average of strain fitness, across strains that have a transposon inserted within that gene. A strain's fitness is the log_2 ratio of abundance at the end of the experiment compared to its abundance at the beginning of the experiment. Fitness < 0 means that the gene was important for fitness and the mutants were less abundant at the end of the experiment than at the beginning. Fitness > 0 means that the gene was detrimental to fitness and that mutants had a growth advantage. If -1 < fitness > 1, the gene has a subtle phenotype that might be statistically significant. Fitness < -2 or fitness > 2 are strong fitness effects.

Group	Condition	b2765	b2777	b0444	b2794	b0406	b0405	b4166
		queD (ygcM)	queE (ygcF)	queC (ybaX)	queF (yqcD)	tgt	queA	queG (yjeS)
motility	outer cut, LB soft agar motility assay	-0.9	-0.2	0	0.2	-4.5	-3.6	-1.2
stress	Benzalkonium Chloride 0.004 mg/ml	-1.1	-0.3	-0.2	-0.7	-3.1	-1.6	-1
motility	outer cut, LB soft agar motility assay	0.5	-0.2	-0.1	-0.1	-2.7	-2.4	-0.9
carbon source	D-Serine (C)	-0.3	-0.1	-0.1	-0.2	-2	-1.3	-1.4
carbon source	D-Serine (C)	-0.2	-0.1	-0.1	0.1	-1.4	-1.2	-1.3
stress	Bacitracin 1 mg/ml	0	-0.1	-0.3	-0.2	-1.6	-1.1	-0.6
stress	2-Furfuraldehyde 0.25 vol%	-0.5	-0.1	0	-0.3	-0.4	-1.4	-0.5
stress	Cisplatin 0.1 mg/ml	0	-0.1	0	-0.7	-1.8	-0.3	-0.4
nitrogen source	Putrescine (N)	0	0	-0.1	0.1	-2.1	-0.6	-0.4
motility	inner cut, LB soft agar motility assay	-0.4	0.2	-0.4	0.1	-1.1	-0.8	-0.4
stress	syringaldehyde 10 mM	-0.1	-0.1	-0.2	0.2	-2	-0.5	-0.3
motility	inner cut, LB soft agar motility assay	-0.3	0.1	0	0.1	-1.4	-0.7	-0.4
nitrogen source	D-Serine (N)	-0.4	0.2	0	0.1	-0.9	-0.8	-0.8
stress	Spectinomycin 0.0125 mg/ml	0.3	0	0.1	-0.6	-1.1	-0.6	-0.3
carbon source	D-Lactate (C)	0.2	0	0.1	0	-1.1	-1.1	-0.6
carbon source	pyruvate (C)	-0.2	-0.1	0	0.2	-0.9	-0.6	-0.5
carbon source	D-Maltose (C)	0.1	0.1	-0.1	-0.1	-1.1	-0.5	-0.5
nitrogen source	Glycine (N)	0.1	0	-0.2	0.2	-0.9	-0.9	-0.4
stress	1-ethyl-3-methylimidazolium chloride 80 mM	0	0.1	0.1	-0.2	-1.6	-0.5	0.1
carbon source	D-Lactate (C)	0.3	-0.1	0.2	-0.1	-0.9	-0.7	-0.6
carbon source	D-Xylose (C)	0.3	0	0.1	0.1	-1.1	-0.8	-0.6
stress	Aluminum chloride 5 mM	-0.1	0.3	0.1	-0.3	-1	-0.6	-0.2
carbon source	D-Xylose (C)	0.1	0	0	0.2	-0.8	-0.7	-0.6
motility	inner cut, LB soft agar motility assay	0.1	0.1	-0.2	0.3	-1.2	-0.5	-0.5
stress	Cisplatin 0.05 mg/ml	0.4	0	0.1	-0.4	-1	-0.6	-0.1
carbon source	D-Glucose-6-Phosphate (C)	0.2	0.1	0	0.2	-1	-0.8	-0.4
motility	inner cut, LB soft agar motility assay	0.4	-0.1	0.1	-0.5	-1	-0.4	0
stress	Chloride 750 mM	0.4	0.1	-0.1	0	-0.8	-0.7	-0.3
stress	Cobalt chloride 0.32 mM	-0.2	0	0	0.1	1.2	0.9	0.6
stress	Nickel (II) chloride 1 mM	0.8	0	-0.1	0.1	1.2	1.3	0.8

Table S4. List of Fe-S proteins in *E. coli*. Relative mRNA expression levels where calculated based on the comparison of the *tgt* mutant to the wild-type strain grown in LB medium (tgt_LB/WT_LB). List obtained from: Iron Sulfur ProteHome (http://biodev.extra.cea.fr/isph/results.aspx?organismid=ECOLI, accessed 06/25/2018) (Estellon et al., 2014) and from Life without Fe–S clusters (Rocha & Dancis, 2016). Significantly up-regulated genes in the *tgt* mutant display the number 1 in the last column, while down-regulated genes display -1.

Gene	Product	log_2(tgt_LB/WT_LB)	significant differentially expressed genes (tgt_LB/WT_LB)
**iscR**	IscR DNA-binding transcriptional dual regulator	2.19	1
**iscU**	scaffold protein for iron-sulfur cluster assembly	1.75	1
**iscA**	iron-sulfur cluster assembly protein	1.63	1
**nfuA**	iron-sulfur cluster scaffold protein	1.50	1
**erpA**	essential respiratory protein A	1.44	1
**fumA**	fumarase A	1.20	1
**fdx**	oxidized ferredoxin // "reduced ferredoxin"	1.04	1
**fadH**	DIENOYLCOAREDUCT-MONOMER	0.82	1
**sdhB**	succinate:quinone oxidoreductase, iron-sulfur cluster binding protein	0.78	1
**acnB**	bifunctional aconitate hydratase 2 and 2-methylisocitrate dehydratase	0.69	1
**nuoF**	NADH:ubiquinone oxidoreductase, chain F	0.69	1
**ilvD**	dihydroxy acid dehydratase	0.59	1
**sdaA**	L-serine deaminase I	0.45	1
**nuoE**	NADH:ubiquinone oxidoreductase, chain E	0.67	0
**rfbD**	DTDPDEHYRHAMREDUCT-MONOMER	0.62	0
**tdcG**	L-serine deaminase III	0.57	0
**fnr**	FNR DNA-binding transcriptional dual regulator	0.54	0
**acnA**	aconitate hydratase 1	0.47	0
**preA**	NADH-dependent dihydropyrimidine dehydrogenase subunit	0.46	0
**rsxC**	member of SoxR-reducing complex	0.45	0
**yfaE**	2Fe-2S cluster-containing protein involved in diferric-tyrosyl radical cofactor maintenance	0.45	0
**nuoB**	NADH:ubiquinone oxidoreductase, chain B	0.42	0
**queE**	conserved protein	0.39	0
**yggW**	predicted oxidoreductase	0.38	0
**gltB**	glutamate synthase, large subunit	0.33	0
**paaE**	ring 1,2-phenylacetyl-CoA epoxidase, reductase subunit	0.29	0
**hcaE**	3-phenylpropionate dioxygenase, α subunit	0.28	0
**bisC**	biotin sulfoxide reductase	0.28	0
**nuoI**	NADH:ubiquinone oxidoreductase, chain I	0.27	0
**ispH**	1-hydroxy-2-methyl-2-(E)-butenyl 4-diphosphate reductase	0.27	0

**miaB**	isopentenyl-adenosine A37 tRNA methylthiolase	0.26	0
**ydiJ**	predicted FAD-linked oxidoreductase	0.25	0
**rlmN**	23S rRNA m²A2503 methyltransferase and tRNA m²A37 methyltransferase	0.25	0
**nsrR**	NsrR DNA-binding transcriptional repressor	0.23	0
**lipA**	lipoyl synthase	0.19	0
**sdaB**	L-serine deaminase II	0.19	0
**fdoG**	formate dehydrogenase-O, α subunit	0.18	0
**ttcA**	tRNA C32 thiolase	0.15	0
**ycbX**	protein involved in base analog detoxification	0.10	0
**fdoH**	formate dehydrogenase-O, β subunit	0.10	0
**aslB**	predicted anaerobic sulfatase maturation enzyme	0.09	0
**nuoG**	NADH:ubiquinone oxidoreductase, chain G	0.08	0
**mutY**	adenine glycosylase; G.C --> T.A transversions	0.08	0
**epmB**	lysine 2,3-aminomutase	0.06	0
**rsxB**	member of SoxR-reducing complex	0.06	0
**yeaW**	2Fe-2S cluster-containing protein	0.04	0
**hcaC**	3-phenylpropionate dioxygenase, predicted ferredoxin subunit	0.01	0
**nadA**	quinolinate synthase	0.00	0
**hemN**	coproporphyrinogen III dehydrogenase	0.00	0
**grxD**	glutaredoxin 4	-0.07	0
**pflA**	pyruvate formate-lyase activating enzyme	-0.10	0
**ispG**	EG10370-MONOMER	-0.10	0
**ydhX**	predicted 4Fe-4S ferredoxin-type protein	-0.12	0
**mug**	stationary phase mismatch/uracil DNA glycosylase	-0.12	0
**thiC**	THIC-MONOMER	-0.12	0
**ahpF**	alkyl hydroperoxide reductase, AhpF component	-0.14	0
**glcF**	glycolate oxidase, predicted iron-sulfur subunit	-0.14	0
**xdhC**	xanthine dehydrogenase, Fe-S subunit	-0.15	0
**ygiQ**	conserved protein	-0.17	0
**yeiL**	YeiL DNA-binding transcriptional activator	-0.18	0
**rimO**	ribosomal protein S12 D88 methylthiotransferase	-0.19	0
**yidL**	predicted DNA-binding transcriptional regulator	-0.20	0
**hyfH**	hydrogenase 4, component H	-0.20	0
**rlmC**	23S rRNA m⁵U747 methyltransferase	-0.21	0
**bfd**	bacterioferritin-associated ferredoxin	-0.21	0
**leuC**	LeuC	-0.21	0
**gltD**	glutamate synthase, small subunit	-0.22	0
**pflC**	probable pyruvate formate lyase 2 activating enzyme	-0.22	0
**yeaX**	predicted oxidoreductase	-0.24	0
**preT**	NADH-dependent dihydropyrimidine dehydrogenase subunit	-0.24	0
**ygfS**	predicted oxidoreductase, 4Fe-4S ferredoxin-type subunit	-0.26	0
**bioB**	biotin synthase	-0.27	0
**torZ**	trimethylamine N-oxide reductase, TorZ subunit	-0.27	0

**ydeP**	acid resistance protein	-0.31	0
**thiH**	2-iminoacetate synthase	-0.32	0
**yfhL**	-0.36	0	
**phnJ**	carbon-phosphorus lyase core complex, PhnJ subunit	-0.38	0
**nth**	endonuclease III	-0.38	0
**glpC**	anaerobic glycerol-3-phosphate dehydrogenase subunit C	-0.39	0
**dinG**	ATP-dependent helicase	-0.39	0
**paoA**	aldehyde dehydrogenase, Fe-S subunit	-0.39	0
**nrdG**	anaerobic ribonucleoside-triphosphate reductase-activating protein	-0.43	0
**narY**	nitrate reductase Z, β subunit	-0.44	0
**cysI**	sulfite reductase, hemoprotein subunit	-0.47	0
**ynfG**	putative selenate reductase, predicted Fe-S subunit	-0.48	0
**narZ**	nitrate reductase Z, α subunit	-0.49	0
**sufA**	Fe-S transport protein in Fe-S cluster assembly	-0.50	0
**sufB**	SufB component of SufBCD Fe-S cluster scaffold complex	-0.55	0
**ygcO**	predicted 4Fe-4S cluster-containing protein	-0.55	0
**fhuF**	hydroxamate siderophore iron reductase	-0.56	0
**hycF**	formate hydrogenlyase complex iron-sulfur protein	-0.60	0
**ydeM**	predicted anaerobic sulfatase maturation enzyme	-0.66	0
**fdnH**	formate dehydrogenase N, β subunit	-0.87	0
**torA**	trimethylamine N-oxide reductase, catalytic subunit	-0.43	-1
**ybhJ**	predicted hydratase	-0.45	-1
**hyfA**	hydrogenase 4, component A	-0.47	-1
**hycE**	hydrogenase 3, large subunit	-0.50	-1
**hycB**	hydrogenase 3, Fe-S subunit	-0.51	-1
**hydN**	putative electron transport protein HydN	-0.51	-1
**xdhD**	fused predicted xanthine/hypoxanthine oxidase: molybdopterin-binding subunit and Fe-S binding subunit	-0.53	-1
**hycG**	hydrogenase 3 and formate hydrogenlyase complex, HycG subunit	-0.58	-1
**hyaA**	hydrogenase 1, small subunit	-0.58	-1
**ttdA**	L-tartrate dehydratase, α subunit	-0.60	-1
**ydiT**	predicted 4Fe-4S ferredoxin-type protein	-0.61	-1
**queG**	epoxyqueuosine reductase	-0.62	-1
**ttdB**	L-tartrate dehydratase, β subunit	-0.62	-1
**hyfI**	hydrogenase 4, small subunit	-0.62	-1
**hyfG**	hydrogenase 4, large subunit	-0.63	-1
**pfo**	pyruvate:flavodoxin oxidoreductase	-0.65	-1
**prpD**	G6199-MONOMER	-0.66	-1
**yhaM**	conserved protein	-0.68	-1
**ygfT**	fused predicted oxidoreductase, Fe-S subunit and nucleotide-binding subunit	-0.71	-1
**glpA**	anaerobic glycerol-3-phosphate dehydrogenase subunit A	-0.78	-1
**fixX**	putative ferredoxin possibly involved in anaerobic carnitine metabolism	-0.78	-1
**ygfK**	predicted oxidoreductase, Fe-S subunit	-0.79	-1

**ynfF**	putative selenate reductase, oxidoreductase subunit	-0.81	-1
**frdB**	fumarate reductase iron-sulfur protein	-0.85	-1
**yjiL**	-0.87	-1	
**ykgF**	predicted amino acid dehydrogenase with NAD(P)-binding domain and ferridoxin-like domain	-0.95	-1
**fumB**	fumarase B	-0.96	-1
**soxR**	SoxR DNA-binding transcriptional dual regulator	-1.00	-1
**ydhV**	predicted oxidoreductase	-1.00	-1
**moaA**	GTP 3',8'-cyclase	-1.09	-1
**yccM**	predicted 4Fe-4S membrane protein	-1.12	-1
**ynfE**	putative selenate reductase, oxidoreductase subunit	-1.14	-1
**yjiM**	hypothetical protein	-1.14	-1
**hcr**	G6456-MONOMER	-1.19	-1
**hcp**	hybrid-cluster protein	-1.46	-1
**ybiY**	predicted pyruvate formate lyase activating enzyme	-1.55	-1
**fdhF**	formate dehydrogenase H	-1.60	-1
**napH**	ferredoxin-type protein	-1.64	-1
**nrfC**	formate-dependent nitrite reductase, 4Fe-4S subunit	-1.67	-1
**hybA**	hydrogenase 2 - [Fe-S] binding, ferredoxin-type component HybA	-1.69	-1
**napG**	ferredoxin-type protein	-1.73	-1
**ydhY**	predicted 4Fe-4S ferredoxin-type protein	-1.83	-1
**aegA**	putative oxidoreductase, Fe-S subunit	-1.84	-1
**yhcC**	predicted Fe-S oxidoreductase	-1.95	-1
**dmsB**	dimethyl sulfoxide reductase, chain B	-2.07	-1
**ydjY**	predicted protein	-2.14	-1
**ysaA**	predicted hydrogenase, 4Fe-4S ferredoxin-type component	-2.26	-1
**fdnG**	formate dehydrogenase N, α subunit	-2.32	-1
**hybO**	hydrogenase 2, small subunit	-2.36	-1
**narH**	nitrate reductase A, β subunit	-2.43	-1
**yjjW**	predicted pyruvate formate lyase activating enzyme	-2.48	-1
**napA**	large subunit of periplasmic nitrate reductase, molybdoprotein	-2.68	-1
**dmsA**	dimethyl sulfoxide reductase, chain A	-3.07	-1
**napF**	ferredoxin-type protein	-3.42	-1
**nirD**	nitrite reductase, small subunit	-3.66	-1
**nirB**	nitrite reductase, large subunit	-4.00	-1
**narG**	nitrate reductase A, α subunit	-4.28	-1

References

Baek, J. H., Han, M.-J., Lee, S. Y., & Yoo, J.-S. (2009). Transcriptome and proteome analyses of adaptive responses to methyl methanesulfonate in *Escherichia coli* K-12 and *ada* mutant strains. *BMC Microbiol*, *9*, 186. https://doi.org/10.1186/1471-2180-9-186

Berardini, M., Foster, P. L., & Loechler, E. L. (1999). DNA polymerase II (*polB*) is involved in a new DNA repair pathway for DNA interstrand cross-links in *Escherichia coli*. *J Bacteriol*, *181*(9), 2878–2882. https://doi.org/10.1128/JB.181.9.2878-2882.1999

Blattner, F. R., Plunkett, G., Bloch, C. A., Perna, N. T., Burland, V., Riley, M., Collado-Vides, J., Glasner, J. D., Rode, C. K., Mayhew, G. F., Gregor, J., Davis, N. W., Kirkpatrick, H. A., Goeden, M. A., Rose, D. J., Mau, B., & Shao, Y. (1997). The complete genome sequence of *Escherichia coli* K-12. *Science (New York, N.Y.)*, *277*(5331), 1453–1462. https://doi.org/10.1126/science.277.5331.1453

Estellon, J., Ollagnier De Choudens, S., Smadja, M., Fontecave, M., & Vandenbrouck, Y. (2014). An integrative computational model for large-scale identification of metalloproteins in microbial genomes: A focus on iron-sulfur cluster proteins. *Metallomics*, *6*(10), 1913–1930. https://doi.org/10.1039/c4mt00156g

Ezraty, B., & Barras, F. (2016). The “liaisons dangereuses” between iron and antibiotics. *FEMS Microbiol Rev*, *40*(3), 418–435. https://doi.org/10.1093/femsre/fuw004

Grenier, F., Matteau, D., Baby, V., & Rodrigue, S. (2014). Complete Genome Sequence of Escherichia coli BW25113. *Genome Announcements*, *2*(5). https://doi.org/10.1128/genomeA.01038-14

Guzman, L. M., Belin, D., Carson, M. J., & Beckwith, J. (1995). Tight regulation, modulation, and high-level expression by vectors containing the arabinose PBAD promoter. *Journal of Bacteriology*, *177*(14), 4121–4130. https://doi.org/10.1128/jb.177.14.4121-4130.1995

Hutinet, G., Swarjo, M. A., & de Crécy-Lagard, V. (2017). Deazaguanine derivatives, examples of crosstalk between RNA and DNA modification pathways. *RNA Biol*, *14*(9), 1175–1184. https://doi.org/10.1080/15476286.2016.1265200

Hutinet, G., Kot, W., Cui, L., Hillebrand, R., Balamkundu, S., Gnanakalai, S., Neelakandan, R., Carstens, A. B., Fa Lui, C., Tremblay, D., Jacobs-Sera, D., Sassanfar, M., Lee, Y.-J., Weigele, P., Moineau, S., Hatfull, G. F., Dedon, P. C., Hansen, L. H., & de Crécy-Lagard, V. (2019). 7-Deazaguanine modifications protect phage DNA from host restriction systems. *Nature Communications*, *10*(1), 5442. https://doi.org/10.1038/s41467-019-13384-y

Igloi, G. L., & Kössel, H. (1985). Affinity electrophoresis for monitoring terminal phosphorylation and the presence of queuosine in RNA. Application of polyacrylamide containing a covalently bound boronic acid. *Nucleic Acids Res*, *13*(19), 6881-98. https://doi.org/10.1093/nar/13.19.6881

Keyer, K., & Imlay, J. A. (1996). Superoxide accelerates DNA damage by elevating free-iron levels. *Proc Natl Acad Sci U S A*, *93*(24), 13635–13640. https://doi.org/10.1073/pnas.93.24.13635

Mendoza-Chamizo, B., Løbner-Olesen, A., & Charbon, G. (2018). Coping with reactive oxygen species to ensure genome stability in *Escherichia coli*. *Genes*, *9*(11). https://doi.org/10.3390/genes9110565

Mielecki, D., & Grzesiuk, E. (2014). Ada response - a strategy for repair of alkylated DNA in bacteria. *FEMS Microbiol Lett*, *355*(1), 1–11. https://doi.org/10.1111/1574-6968.12462

Miller, J. H. (1972). *Experiments in molecular genetics*. Cold Spring Harbor, N. Y. Cold Spring Harbor Laboratory.

Phillips, G., Grochowski, L. L., Bonnett, S., Xu, H., Bailly, M., Blaby-Haas, C., el Yacoubi, B., Iwata-Reuyl, D., White, R. H., & de Crécy-Lagard, V. (2012). Functional promiscuity of the COG0720 family. *ACS Chemical Biology*. https://doi.org/10.1021/cb200329f

Price, M. N., Wetmore, K. M., Waters, R. J., Callaghan, M., Ray, J., Liu, H., Kuehl, J. v., Melnyk, R. A., Lamson, J. S., Suh, Y., Carlson, H. K., Esquivel, Z., Sadeeshkumar, H., Chakraborty, R., Zane, G. M., Rubin, B. E., Wall, J. D., Visel, A., Bristow, J., … Deutschbauer, A. M. (2018). Mutant phenotypes for thousands of bacterial genes of unknown function. *Nature*, *557*(7706), 503–509. https://doi.org/10.1038/s41586-018-0124-0

Rocha, A. G., & Dancis, A. (2016). Life without Fe-S clusters. *Molecular Microbiology*, *99*(5), 821–826. https://doi.org/10.1111/mmi.13273

Roche, B., Huguenot, A., Barras, F., & Py, B. (2015). The iron-binding CyaY and IscX proteins assist the ISC-catalyzed Fe-S biogenesis in *Escherichia coli*. *Molecular Microbiology*, *95*(4), 605–623. https://doi.org/10.1111/mmi.12888

Seaver, L. C., & Imlay, J. A. (2004). Are Respiratory Enzymes the Primary Sources of Intracellular Hydrogen Peroxide? *Journal of Biological Chemistry*, *279*(47), 48742–48750. https://doi.org/10.1074/jbc.M408754200

Seo, S. W., Kim, D., Latif, H., O'Brien, E. J., Szubin, R., & Palsson, B. O. (2014). Deciphering *fur* transcriptional regulatory network highlights its complex role beyond iron metabolism in *Escherichia coli*. *Nat Commun*, *5*, 4910. https://doi.org/10.1038/ncomms5910

Sikora, A., Maciejewska, A. M., Poznański, J., Pilżys, T., Marcinkowski, M., Dylewska, M., Piwowarski, J., Jakubczak, W., Pawlak, K., & Grzesiuk, E. (2015). Effects of changes in intracellular iron pool on AlkB-dependent and AlkB-independent mechanisms protecting *E. coli* cells against mutagenic action of alkylating agent. *Mutat Res, Fundam Mol Mech Mutagen*, *778*, 52–60. https://doi.org/10.1016/j.mrfmmm.2015.05.009

Taghizadeh, K., McFaline, J. L., Pang, B., Sullivan, M., Dong, M., Plummer, E., & Dedon, P. C. (2008). Quantification of DNA damage products resulting from deamination, oxidation and reaction with products of lipid peroxidation by liquid chromatography isotope dilution tandem mass spectrometry. *Nat Protoc*, *3*(8), 1287–1298. https://doi.org/10.1038/nprot.2008.119

Thiaville, J. J., Kellner, S. M., Yuan, Y., Hutinet, G., Thiaville, P. C., Jumpathong, W., Mohapatra, S., Brochier-Armanet, C., Letarov, A. v, Hillebrand, R., Malik, C. K., Rizzo, C. J., Dedon, P. C., & de Crécy-Lagard, V. (2016). Novel genomic island modifies DNA with 7-deazaguanine derivatives. *Proc Natl Acad Sci U S A*, *113*(11), E1452-9. https://doi.org/10.1073/pnas.1518570113

van Houten, B., Santa-Gonzalez, G. A., & Camargo, M. (2018). DNA repair after oxidative stress: current challenges. *Curr Opin Toxicol*, *7*, 9–16. https://doi.org/10.1016/j.cotox.2017.10.009

Vinella, D., Loiseau, L., de Choudens, S. O., Fontecave, M., & Barras, F. (2013). In vivo [Fe-S] cluster acquisition by IscR and NsrR, two stress regulators in Escherichia coli. *Molecular Microbiology*, *87*(3), 493–508. https://doi.org/10.1111/mmi.12135

Yuan, Y., Zallot, R., Grove, T. L., Payan, D. J., Martin-Verstraete, I., Šepić, S., Balamkundu, S., Neelakandan, R., Gadi, V. K., Liu, C.-F., Swairjo, M. A., Dedon, P. C., Almo, S. C., Gerlt, J. A., & de Crécy-Lagard, V. (2019). Discovery of novel bacterial queuine salvage enzymes and pathways in human pathogens. *Proc Natl Acad Sci U S A*, *116*(38), 19126–19135. https://doi.org/10.1073/pnas.1909604116

Zallot, R., Yuan, Y., & de Crécy-Lagard, V. (2017). The *Escherichia coli* COG1738 member YhhQ is involved in 7-cyanodeazaguanine ($preQ_0$) transport. *Biomolecules*, *7*(1), 1–13. https://doi.org/10.3390/biom7010012

Zhang, X., & Bremer, H. (1995). Control of the *Escherichia coli rrnB* P1 promoter strength by ppGpp. *J Biol Chem*, *270*(19), 11181–11189. https://doi.org/10.1074/jbc.270.19.11181
